# Sex differences in the response of the alveolar macrophage proteome to treatment with exogenous surfactant protein-A

**DOI:** 10.1186/1477-5956-10-44

**Published:** 2012-07-23

**Authors:** David S Phelps, Todd M Umstead, Joanna Floros

**Affiliations:** 1 Center for Host defense, Inflammation, and Lung Disease(CHILD) Research and Department of Pediatrics, The Pennsylvania State University College of Medicine, Hershey, PA, 17033, USA; 2 Department of Obstetrics and Gynecology, The Pennsylvania State University College of Medicine, Hershey, PA, 17033, USA

**Keywords:** Pneumonia, Chaperone, Actin, Nrf2, Innate immunity, Phagocytosis, Oxidative stress, Estrogen, SP-A, Lung

## Abstract

**Background:**

Male wild type (WT) C57BL/6 mice are less capable of clearing bacteria and surviving from bacterial pneumonia than females. However, if an oxidative stress (acute ozone exposure) occurs before infection, the advantage shifts to males who then survive at higher rates than females. We have previously demonstrated that survival in surfactant protein-A (SP-A) knockout (KO) mice compared to WT was significantly reduced. Because the alveolar macrophage (AM) is pivotal in host defense we hypothesized that SP-A and circulating sex hormones are responsible for these sex differences. We used 2D-DIGE to examine the relationship of sex and SP-A on the AM proteome. The role of SP-A was investigated by treating SP-A KO mice with exogenous SP-A for 6 and 18 hr and studying its effects on the AM proteome.

**Results:**

We found: 1) less variance between KO males and females than between the WT counterparts by principal component analysis, indicating that SP-A plays a role in sex differences; 2) fewer changes in females when the total numbers of significantly changing protein spots or identified whole proteins in WT or 18 hr SP-A-treated males or females were compared to their respective KO groups; 3) more proteins with functions related to chaperones or protease balance and Nrf2-regulated proteins changed in response to SP-A in females than in males; and 4) the overall pattern of SP-A induced changes in actin-related proteins were similar in both sexes, although males had more significant changes.

**Conclusions:**

Although there seems to be an interaction between sex and the effect of SP-A, it is unclear what the responsible mechanisms are. However, we found that several of the proteins that were expressed at significantly higher levels in females than in males in WT and/or in KO mice are known to interact with the estrogen receptor and may thus play a role in the SP-A/sex interaction. These include major vault protein, chaperonin subunit 2 (beta) (CCT2), and Rho GDP alpha dissociation inhibitor. We conclude that sex differences exist in the proteome of AM derived from male and female mice and that SP-A contributes to these sex differences.

## Introduction

A growing number of studies have described sex differences in the incidence and severity of pulmonary diseases [1-4]. Perhaps the most thoroughly characterized of these are COPD [5-8] and asthma [9-11]. Sex differences have also been reported in the incidence of some pneumonias, although these data are not as straightforward [12-15]. However, despite this increasing recognition of sex differences serious gaps remain in our understanding of the responsible mechanisms.

A number of laboratories including our own, have employed animal models to study sex differences in pulmonary disease [16]. These studies include models for COPD, asthma [17,18], fibrosis [19], pneumonia [20-22], and others. Our studies with mice infected with *Klebsiella pneumoniae* found sex differences in the susceptibility to infection (males more affected than females). However, when mice were infected after being exposed to ozone, the sex differences went in the opposite direction (females more affected than males) [20]. Ozone-exposed *K. pneumoniae*-infected females showed an excessive lung inflammatory response compared to their male counterparts [22]. In the absence of ozone exposure, males had higher levels of bacterial dissemination compared to females, whereas in ozone-exposed females, the bacterial clearance in the lung was decreased more than in males [23]. Circulating sex hormones (estrogen, dihydrotestosterone; DHT) were recently shown to play a role in these survival differences [24]. Comparison of *K. pneumoniae*-infected mice lacking surfactant protein A (SP-A)(SP-A knockout; KO) to wild type (WT) mice on the C57BL/6 genetic background showed a poorer survival rate [21] with the sex differences persisting in the presence of ozone-induced oxidative stress.

SP-A is a collagenous lectin or collectin that is known to influence host-defense function in the lung in a number of ways [25,26]. These include its ability to function as an opsonin aiding in the clearance of a variety of pathogens by alveolar macrophages (AM), to influence the production of inflammatory mediators by lung immune cells, and to alter the expression profile of the bronchoalveolar lavage (BAL) proteome in the lung [27,28].

The AM plays a pivotal role in innate host defense function. AM from male mice have been shown to have significantly higher phagocytic indices in response to infection with *K. pneumoniae* than females after exposure to ozone [20]. Similar observations have been made for AM from SP-A KO mice, except that the phagocytic indices were lower than those from WT AM, indicating that macrophage function was impaired in the absence of SP-A [21]. Recently we showed that SP-A has a significant impact on the AM proteome in males [29].

In an effort to better understand the basis for the differences in macrophage function in the presence or absence of SP-A and between males and females we studied the AM proteome. The proteome of AM from females as well as a comparison of the proteome from male [29] and female WT mice and KO mice that received exogenous SP-A was studied using two-dimensional difference gel electrophoresis (2D-DIGE) coupled with MALDI-ToF/ToF, and the Ingenuity Pathways Analysis program.

## Results

### Overview

The proteomics study of AM from male [29] and female (present study) WT and KO mice was performed as a single proteomics experiment, thereby allowing us to directly compare the data. The data for the male AM were previously analyzed and published along with a proof of principle experiment demonstrating SP-A effects on the actin cytoskeleton [29]. In this report the focus is on: a) the study of the data from female AM; and b) the comparison of changes in the female and male AM proteomes in response to exogenous SP-A treatment of SP-A KO mice.

### BAL and cells

Cell pellets were examined from all BAL samples and underwent total and differential cell counts to exclude the possibility that any of the mice had underlying infectious or inflammatory processes. No evidence of inflammation or infection was seen in any of the mice. There were no significant differences in total cell counts and all BAL samples consisted of > 95% macrophages (data not shown). Protein content was the same in all samples (data not shown) and identical amounts of protein (20 μg) from each sample were loaded on all analytical gels.

### 2D-DIGE results

#### Overview

**Figure 1:**
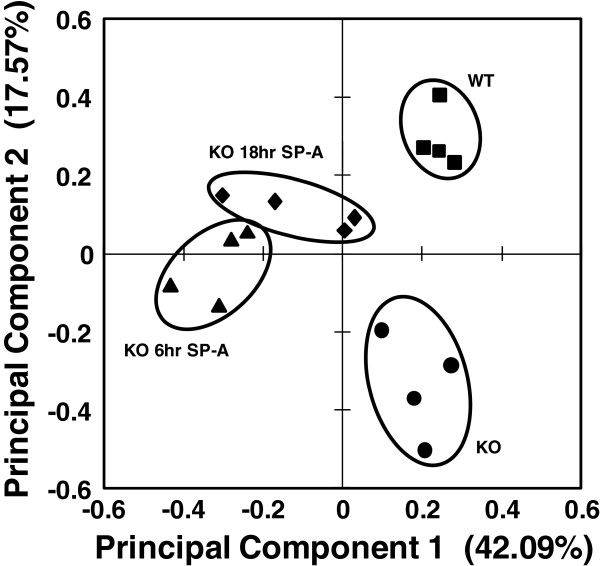
**Principal component analysis of different treatment groups.** A plot of the principal component analysis for the 172 significant (ANOVA, p < 0.05) protein spots is shown. The markers represent the weighted average for the first two principal components for the 172 proteins for each individual in each of the groups: SP-A knockout (KO) (·), SP-A knockout treated with SP-A for 6 hr (KO 6 hr SP-A) (▲), SP-A knockout treated with SP-A for 18 hr (KO 18 hr SP-A) (♦), and wild type (WT) (■).

Gels from the 2D-DIGE study were subjected to automatic spot detection using the Progenesis SameSpots program, after which we performed manual editing. This resulted in 791 protein spots that were matched across 2D-DIGE gels from all samples. Statistical analysis was then done on the individual protein spots. Statistical comparisons were done between the experimental groups of each sex (e. g. KO versus WT) and between sexes for each experimental condition (e. g. male KO versus female KO). Principal component analysis (Figure 1) of the 172 gel spots that differed by ANOVA (p > 0.05) offered an overview of the data and showed that the four experimental groups of female mice were relatively well grouped with no overlap between groups. The two steady-state groups (KO and WT) were the best separated and most tightly clustered groups. The two female SP-A-treated (6 hr and 18 hr) groups were more closely associated to one another indicating less variance between these groups during the 6 and 18 hr treatment intervals. This lack of clear separation between some members of these treatment groups may reflect differences in response kinetics among individuals in each group. We then picked all 791 of the individual protein spots from a preparative gel and subjected then to MALDI-ToF/ToF resulting in the identification of 76 distinct proteins (See Additional files 1 and 2 – these have been published previously [29]). The values for all gel spots representing a given protein were added together and additional statistical analysis, including a series of pairwise comparisons, was then done on all identified proteins. In Table 1 all proteins with significant increases and decreases relative to untreated KO protein levels in at least one comparison are indicated and significant changes are noted.

**Table 1:** Significant changes in alveolar macrophage proteins for Female mice (as compared to KO baseline)

**Gel no.**	**Protein name**	**KO 6 hr SP-A ** **Female**	**KO 18 hr SP-A ** **Female**	**WT ** **Female**
5	Alpha-fetoprotein	↑*	**↑**	**↑**
8	Anxa5 protein	**↑**	**↓**	↑*
11	Calpain, small subunit 1	**↑**	↓*	**↓**
12	Capping protein (actin filament) muscle Z-line, alpha 2 (CapZ alpha-2)	↓*	↓*	**↓**
13	Capping protein (actin filament) muscle Z-line, beta isoform a (CapZ beta)	↓*	**↓**	**↓**
24	Eno1 protein (Alpha-enolase)	↑*	**↑**	**↑**
25	Eukaryotic translation initiation factor 5A	**↑**	↑*	**↑**
26	Ezrin	↓*	**↓**	**↓**
29	Ferritin light chain 1	**↓**	**↓**	↓*
30	Gamma-actin	**↓**	↓*	**↓**
34	Heat shock protein 1, beta (HSP90AB1)	↓*	**↓**	↓*
35	Heat shock protein 5 precursor (GRP78)	**↑**	**↑**	↓*
40	Heme-binding protein	↓*	**↓**	↓*
41	Heterogeneous nuclear ribonucleoprotein K	↓*	**↓**	**↓**
45	Keratin complex 2, basic, gene 8	**↓**	**↑**	↑*
52	Nucleophosmin 1	**↓**	↓*	↓*
53	p50b	↓*	**↓**	**↓**
55	Prolyl 4-hydroxylase, beta polypeptide precursor	↑*	↑*	**↓**
56	Proteasome (prosome, macropain) 28 subunit, alpha	↑*	**↑**	↑*
61	Purine nucleoside phosphorylase	↑*	**↑**	↑*
64	Rho GDP dissociation inhibitor (GDI) alpha	**↑**	↑*	↑*
67	Stathmin	**↑**	↑*	↑*
74	Vacuolar adenosine triphosphatase subunit B	↑*	**↑**	**↑**
76	Vimentin	**↓**	↓*	**↓**
	**Total significant changes**	7↓*, 6↑*	5↓*, 4↑*	5↓*, 6↑*

Comparison of mean normalized volumes (see Additional file 3) for proteins from female KO mice to KO 6 hr SP-A, KO 18 hr SP-A, and WT mice. Increased compared to KO(↑), decreased compared to KO(↓), determined to be significant (p < 0.05) by *t*-test (*). Changes in all proteins are given in Additional file 4 - Table F.

#### Identification and categorization of identified proteins

MALDI-ToF/ToF was used to analyze all 791 detected gel spots and resulted in the identification of 290 spots (comprising 76 unique proteins) with confidence intervals > 95% and ProteinPilot scores of > 61. These have been described in a previous publication [29] and the information about the proteins and a reference gel are included as supplementary material (see Additional files 1 and 2). Some of these identified proteins consisted of a single gel spot and others consisted of multiple spots representing isoforms of a specific protein. The values of all spots for a given protein were added together and statistical analysis done on the combined value. Normalized volumes for all identified proteins are given and values were compared between female groups and significant differences are listed in Additional file 3.

As in previous publications and other types of samples (BAL proteins, plasma proteins) we used different approaches [27-30] to assess the function of the 76 identified proteins and their biological relevance to AM function. Some functional information was provided by analyzing the data with the Ingenuity Pathways Analysis (IPA) program. These analyses identified proteins involved in several processes: 1) regulation of actin-based motility (p = 6.85E-08); 2) RhoA signaling (p = 3.98E-07); 3) actin cytoskeleton signaling (p = 3.35E-06); 4) Fcγ receptor-mediated phagocytosis (p = 3.36E-06); and 5) clathrin-mediated endoctyosis signaling (p = 6.11E-05), comprising the five top canonical pathways. In addition the Nrf2-mediated oxidative stress response (p = 1.09E-04) was prominent among the processes implicated by IPA. Furthermore, we employed a manual curation approach, emphasizing findings from the literature related to the lung and macrophages, where available. These approaches strongly implicated motility, phagocytosis, actin signaling, RhoA signaling, and endocytosis (which we retained as a single functional group of “actin-related/cytoskeletal proteins” rather than dividing it into 5 subgroups) as the most involved cellular processes and included 38 of the 76 proteins identified in our study (Additional file 1[31-99]). Given the role of the AM as a mobile phagocyte, this was anticipated and the identification of a substantial subset of identified proteins involved in these processes indicates that SP-A plays a pivotal role in these macrophage functions.

The Nrf2-related protein category included 21 of our identified proteins. Other major cellular processes implicated by our list of identified proteins included regulation of inflammation (20 proteins), protease balance/chaperone function (19 proteins), and regulatory/differentiative processes (8 proteins). These functional groups also represent important facets of AM biology and thereby constitute a valuable tool in assessing macrophage function in the presence or absence of SP-A. Protease/chaperone function may be very important in the repair of damage to lung tissue and proteins potentially resulting from exposure to noxious material, pathogens, or other danger signals. Similarly, the regulation of inflammatory processes is extremely important for innate immune processes, with dysregulation of inflammation playing a central role in many pulmonary disease processes. Finally, the profound differences between circulating blood monocytes and the AM [100-102] and between macrophages that have undergone different modes of activation [100,103], indicate the presence of an active regulatory mechanism directing the differentiation of macrophages from monocytes and their activation in various directions. The functional groups to which each protein was assigned are listed in Additional file 1. Note that some proteins are included in more than one group.

### AM proteome differences under baseline conditions and in response to SP-A

a) *Comparison between female WT and KO mice:* When AM from WT female mice were compared to AM from KO females (Table 1) 5 proteins were at significantly lower levels in the WT mice versus KO. There were 6 proteins that had significantly higher levels of expression in WT animals than in KO. When KO mice were treated with exogenous SP-A there were changes in levels of protein expression that approximate the WT profile indicating that SP-A treatment tends to restore the WT phenotype. At 6 hours after treatment there were 7 proteins expressed at significantly lower levels than the KO mice and 6 that were expressed at higher levels than in KO. By 18 hours this trend persisted and 5 proteins were at lower levels and 4 at higher levels resulting in a picture similar to the differences between WT and KO described above.

**Figure 2:**
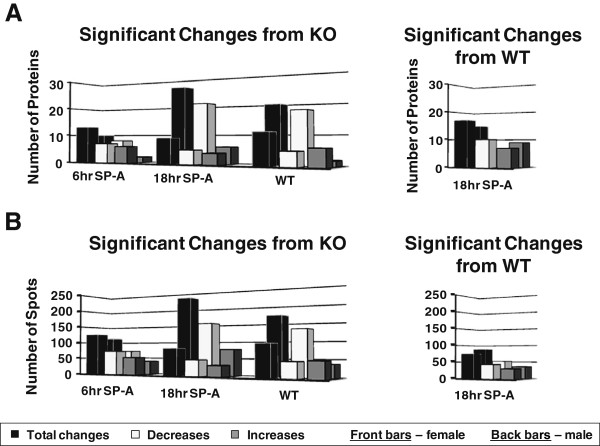
**Summary of significant changes in whole proteins and gel spots.** In Panel **A** the graphs depict the number of significant changes in identified proteins (from a total of 76) compared to KO in macrophages from female mice. The black bar represents the total significant changes, the white bars represent significant decreases relative to KO, and the gray bars represent significant increases relative to KO. The histograms on the right depict significant changes between WT and 18 hr SP-A treated mice. Bars in the front are the values from females (present study) and bars in the back are values from males that we have previously published [29] and are shown here for comparison purposes. In Panel **B** similar comparisons are shown for the gel spots.

The significant changes in the expression of identified proteins are summarized in Figure 2A and in Table 1. A complete list of all proteins showing how they change is in Additional file 4 – Table F. A similar pattern was seen when significant changes in individual gel spots are summarized (Figure 2B). These summary diagrams show the differences between the comparisons in female mice (front bars) and from male mice (back bars) [29]. There were many more significant changes in the males, especially in the 18 hr SP-A versus KO and WT versus KO comparisons. Furthermore, decreases relative to the KO mice predominate in the males, while females had roughly equal numbers of decreasing and increasing proteins/spots.

The comparisons above demonstrate that 18 hr after SP-A treatment of KO mice many aspects of the female AM proteome resembled those seen in WT mice. Statistical comparisons of the 18 hr point with WT mice (right hand panels of Figure 2) showed that the number of significant differences between the 18 hr point and WT were more similar in males and females than the differences observed in the comparisons versus KO mice shown in the left hand panels of Figure 2.

**Figure 3:**
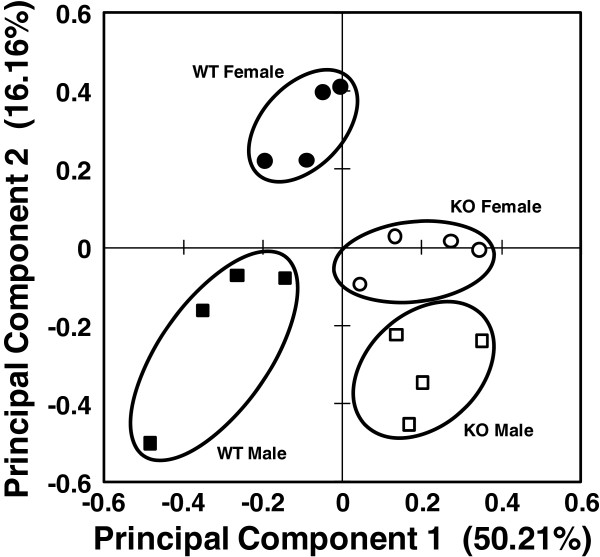
**Principal component analysis of male and female baseline groups.** A plot of the principal component analysis for the 218 significant (ANOVA, p < 0.05) protein spots differing between females and males is shown. The markers represent the weighted average for the first two principal components for the 218 proteins for each individual in each of the groups: Female WT (·), male WT (■), female KO (○), male KO (□).

**Figure 4:**
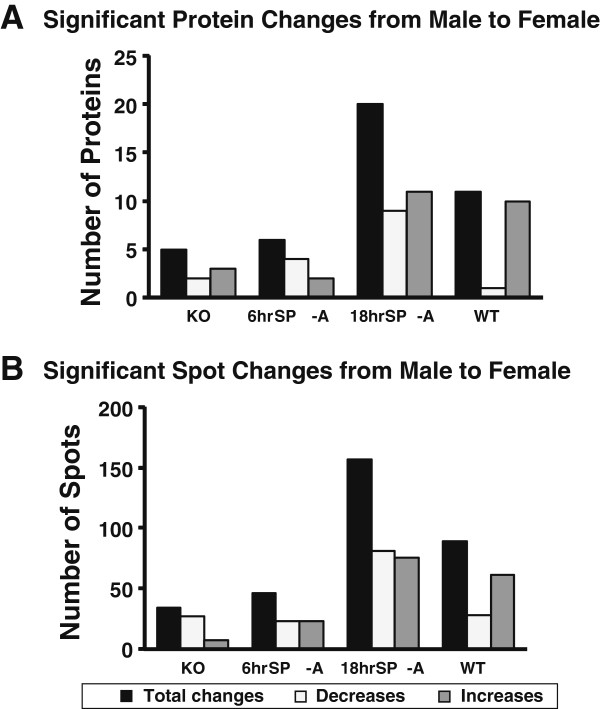
**Summary of significant sex differences in changes in whole proteins and gel spots.** In Panel **A** the graphs depict the number of significant changes in whole proteins (from a total of 76 proteins) when males are compared to females. The black bars represent the total significant changes, the white bars represent significant decreases in females relative to males, and the gray bars represent significant increases in females relative to males. In Panel **B** similar comparisons are shown summarizing the data from gel spots (from a total of 791 spots).

b) *Differences between males and females:* Comparison of baseline levels of WT and KO mice from each sex (data for male mice were from our previous study [29]) identified 218 gel spots by ANOVA that differed significantly. A principal component analysis of these spots revealed that all groups were fairly well consolidated with no overlap (Figure 3). There appeared to be more of a difference between the male and female WT mice, indicating greater variance between them than between the two KO groups, which were very close to one another. This seems to be in general agreement with the whole protein data for the WT mice (Figure 4A; Table 2) where significant differences were observed for 11 of the 76 identified proteins with 10 of these proteins increased and only 1 decreased in females. Indeed when gel spots were analyzed there were 89 spots that differed significantly in WT AM between male and female mice, including 28 decreases (lower in female) and 61 increases (higher in female) (Figure 4B).

**Table 2:** Significant changes in alveolar macrophage proteins from male to female mice in the same group

**Gel no.**	**Protein name**	**KO**	**KO 6 hr SP-A**	**KO 18 hr SP-A**	**WT**
6	Annexin A2	↓*	**↓**	**↓**	**UN**
7	Annexin A4	**↓**	**↑**	↓*	**↓**
8	Anxa5 protein	**↓**	**↓**	↓*	**↓**
12	Capping protein (actin filament) muscle Z-line, alpha 2 (CapZ alpha-2)	**↓**	**↓**	↓*	**↓**
15	Chaperonin subunit 2 (beta) (CCT2)	**↑**	**↑**	**↑**	↑*
17	Chitinase 3-like 3 precursor (Ym1)	**UN**	**↑**	↑*	↑*
20	Chloride intracellular channel 4 (mitochondrial)	**↓**	**↑**	↓*	**↓**
22	Coactosin-like 1	**↓**	**↓**	↓*	**↓**
33	Guanine deaminase	↑*	↑*	↑*	↑*
34	Heat shock protein 1, beta (HSP90AB1)	↑*	**↑**	↑*	**↑**
35	Heat shock protein 5 precursor (GRP78)	↑*	**↑**	↑*	**↑**
36	Heat shock protein 65 (HSP60)	**↑**	**↑**	↑*	↑*
38	Heat shock protein 90, beta (Grp94), member 1	**↓**	**↑**	↑*	**↑**
40	Heme-binding protein	**UN**	↓*	**UN**	**↓**
49	Major vault protein (MVP)	**↑**	**↑**	↑*	↑*
50	Microtubule-associated protein, RP/EB family, member 1	**↓**	**↓**	↑*	↑*
55	Prolyl 4-hydroxylase, beta polypeptide precursor	↓*	**↓**	**↓**	**↓**
59	Protein disulfide-isomerase A3 precursor	**↑**	↑*	**↑**	**↑**
60	Protein synthesis initiation factor 4A	**↓**	**↑**	↑*	↑*
62	Put. beta-actin (aa 27–375)	**↓**	**↓**	↓*	**↓**
63	Rab GDP dissociation inhibitor beta	**↓**	**↑**	**↑**	↑*
64	Rho GDP dissociation inhibitor (GDI) alpha	**↓**	**↑**	↑*	↑*
69	Tropomodulin 3	**↓**	↓*	↓*	**↓**
70	Tropomyosin 3, gamma	**↓**	**↓**	↓*	**↓**
71	Tubulin, beta 5	**↓**	↓*	**↓**	↓*
75	Valosin-containing protein	**↑**	**↑**	↑*	↑*
76	Vimentin	**↓**	↓*	↓*	**↓**
	**Total significant changes**	2↓*, 3↑*	4↓*, 2↑*	9↓*, 11↑*	1↓*, 10↑*

Comparison of mean normalized volumes (see Additional file 3) for proteins from male to female mice in the same group (KO, KO 6 hr SP-A, KO 18 hr SP-A, and WT). Higher in female (**↑**), lower in female (**↓**), unchanged (UN), determined to be significant (p < 0.05) by *t*-test (*). Changes in all proteins are given in Additional file 5 -Table F.

A very different situation was seen in the KO mice where significant differences between male and female KO mice were observed with 5 proteins, less than half the number of significantly changing proteins observed in WT (n = 11) (Table 2; Figure 4A). Also, there were more than 3 times as many protein spots expressed at significantly higher levels in males (n = 27 gel spots) than in females (n = 7 gel spots)(Figure 4B). Consistent with these findings, in the two SP-A treated groups there were relatively few significant differences (6 proteins) between male and female at 6 hr of treatment, but many more significant changes (20 proteins) in the 18 hr SP-A treatment group (Figure 4A). The trend of significant increases or decreases was similar in both individual protein spots (Figure 4B) and in whole proteins (Figure 4A).

#### Gel map overview of protein changes between males and females of WT and KO AM under baseline conditions

**Figure 5:**
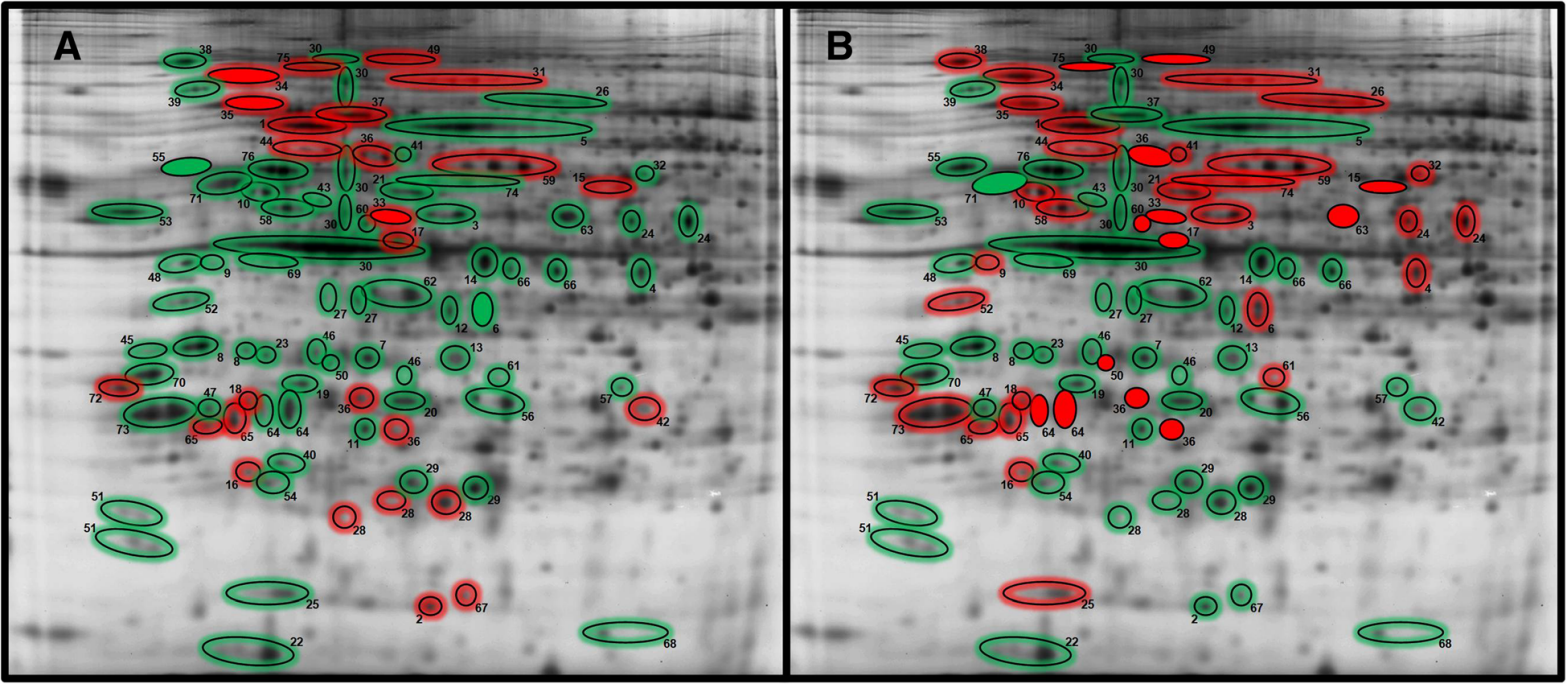
**Sex differences in protein levels in KO and WT mice.** Two identical reference gels (see also Additional file 2) are shown in Figure 5. Protein spots circled in green are expressed at lower levels in females and those with a red border are at higher levels in females. Significant changes are indicated by filled green or red circles. Panel **A** shows the male versus. female comparisons in KO mice and Panel **B** shows the differences in WT mice. The numbers adjacent to each spot indicate the protein and the names are listed below and correspond to those in Additional file 2: 1, 65-kDa macrophage protein; 2, Actin related protein 2/3 complex, subunit 5; 3, Actin-related protein 3; 4, Actr2 protein; 5, Alpha-fetoprotein; 6, Annexin A2; 7, Annexin A4; 8, Anxa5 protein; 9, ArsA arsenite transporter, ATP-binding, homolog 1; 10, Atp5b protein; 11, Calpain, small subunit 1; 12, Capping protein (actin filament) muscle Z-line, alpha 2; 13, Capping protein (actin filament) muscle Z-line, beta isoform a; 14, Cathepsin D precursor; 15, Chaperonin subunit 2 (beta); 16, Chia protein; 17, Chitinase 3-like 3 precursor; 18, Chitinase-related protein MCRP; 19, Chloride intracellular channel 1; 20, Chloride intracellular channel 4; 21, CNDP dipeptidase 2; 22, Coactosin-like 1; 23, EF hand domain containing 2; 24, Eno1 protein (Alpha-enolase); 25, Eukaryotic translation initiation factor 5A; 26, Ezrin; 27, F-actin capping protein alpha-1 subunit; 28, Ferritin heavy chain 1; 29, Ferritin light chain 1; 30, Gamma-actin; 31, Gelsolin precursor; 32, Glucose-6-phosphate dehydrogenase X-linked; 33, Guanine deaminase; 34, Heat shock protein 1, beta; 35, Heat shock protein 5 precursor; 36, Heat shock protein 65; 37, Heat shock protein 8; 38, Heat shock protein 90, beta (Grp94), member 1; 39, Hematopoietic cell specific Lyn substrate 1; 40, Heme-binding protein; 41, Heterogeneous nuclear ribonucleoprotein K; 42, High mobility group 1 protein; 43, Hnrpf protein; 44, Kappa-B motif-binding phosphoprotein; 45, Keratin complex 2, basic, gene 8; 46, Keratin type II; 47, Krt13 protein; 48, Laminin receptor; 49, Major vault protein (MVP); 50, Microtubule-associated protein, RP/EB family, member 1; 51, Myosin light chain, regulatory B-like; 52, Nucleophosmin 1; 53, p50b; 54, Peroxiredoxin 2; 55, Prolyl 4-hydroxylase, beta polypeptide precursor; 56, Proteasome (prosome, macropain) 28 subunit, alpha; 57, Proteasome alpha 1 subunit; 58, Protein disulfide isomerase associated 6; 59, Protein disulfide-isomerase A3 precursor; 60, Protein synthesis initiation factor 4A; 61, Purine nucleoside phosphorylase; 62, Put. beta-actin (aa 27–375); 63, Rab GDP dissociation inhibitor beta; 64, Rho GDP dissociation inhibitor (GDI) alpha; 65, Rho, GDP dissociation inhibitor (GDI) beta; 66, Serine (or cysteine) proteinase inhibitor, clade B, member 1a; 67, Stathmin; 68, Superoxide dismutase 1, soluble; 69, Superoxide dismutase 1, soluble; 70, Tropomyosin 3, gamma; 71, Tubulin, beta 5; 72, Tyrosine 3/tryptophan 5 -monooxygenase activation protein, epsilon polypeptide; 73, Tyrosine 3-monooxygenase/tryptophan 5-monooxygenase activation protein, beta polypeptide; 74, Vacuolar adenosine triphosphatase subunit B; 75, Valosin-containing protein; 76, Vimentin.

Figure 5 shows two identical reference gels with differences in protein spots between males and females outlined with either green when lower in females or red if higher in females, and colored in with solid red or green when sex differences are significant. Panels A and B depict sex differences in KO mice and WT mice, respectively. As shown previously in Table 2 and Figure 4, there were very few significant sex differences in the KO mice (solid green or red in Figure 5A) even though more than twice as many proteins were at lower levels in the female than in their male counterparts. By contrast, in the WT mice (Figure 5B) there were approximately the same numbers of proteins at lower levels in females (green spots) as there were higher in females (red spots); many more of the sex differences were significant, and most of those changes were higher in females.

#### Examples of AM proteome patterns in males and females in response to SP-A treatment

**Figure 6:**
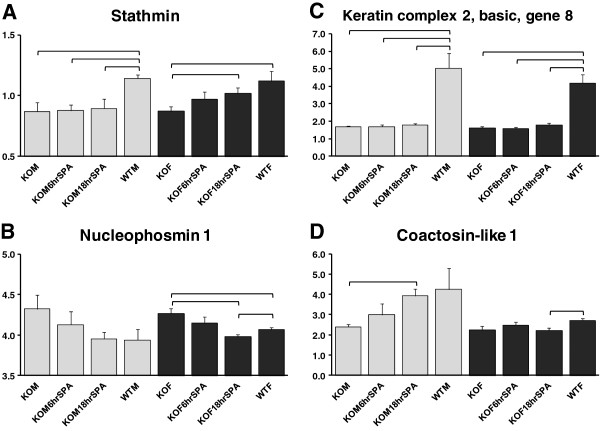
**Examples of protein patterns.** Graphs shown in Panels **A-D** indicate different SP-A-response patterns for: **A**) stathmin; **B**) nucleophosmin 1; **C**) keratin complex 2, basic, gene 8; and **D**) coactosin-like 1. Significant differences (p < 0.05) are indicated by brackets.

Several patterns of changes in response to SP-A were seen. Stathmin (Figure 6A), a protein that regulates microtubule dynamics [104], was significantly higher in WT AM of both sexes than KO AM, and showed increases over KO levels after SP-A treatment in the female but little or no change in the male. With nucleophosmin 1 (Figure 6B), WT levels were lower than KO levels (significantly in females) and there was a time-dependent decrease after SP-A treatment. The levels of keratin complex 2, basic gene 8 (Figure 6C), a protein involved in intermediate filament formation [76], were significantly higher in WT than KO in both sexes, and neither sex showed any apparent change after SP-A treatment. Coactosin-like 1 (Figure 6D), a protein known to bind F-actin and serve as a chaperone for 5-lipoxygenase, increased after SP-A treatment in males but appeared to change little in females (Figure 6D).

#### Sex differences in functional groups

The proteins making up each of our functional groups were studied in order to determine whether there were fundamental differences between male and female WT and KO AM and in response to SP-A treatment in these groups, and whether these differences could explain the differences we have previously reported in pneumonia survival and phagocytic function. The data used for the functional groups below are presented in Additional file 5 Tables A – E (see also Table 2 and Additional file 3). As shown in Table 2 for all of the significantly changed proteins in the WT mice, there were significant sex differences where in almost all cases (10 of 11) female levels exceeded male levels. In contrast, in KO mice only 5 proteins differed significantly between male and female (3 higher in female; 2 in male). By 18 hours after SP-A treatment 20 proteins differed significantly between male and female (11 higher in female; 9 in male). Comparisons of expression levels of all proteins between male and female are shown in Additional file 5- Table F. Next we studied these changes in the individual functional groups.

a) *Protease balance/chaperone function group:*

The most striking sex-related observation in this functional group was the fact that of the 12 significant differences between the sexes in all groups (KO, 6 hr SP-A, 18 hr SP-A, WT) there was only 1 (18 hr SP-A) in which male levels exceeded female levels. Eleven of the 12 significant differences were in chaperones and heat shock proteins and all 11 were significantly higher in females. These results indicate that chaperone function may be increased in females although it is unclear how this would affect cellular stresses. It is also worth noting that of the 12 significant sex differences, half were seen in the 18 hr SP-A treatment group (Additional file 5 – Table A).

In terms of the sex specificity of the SP-A response patterns there were almost twice as many significant differences in males (n = 17)[29] than in females (n = 8)(Additional file 4 – Table A).

b) *Actin-related/cytoskeleton group:* Of the 38 proteins characterized as actin-related/cytoskeletal most of the significant differences between sexes in WT mice were higher in the female (7 out of 8). In contrast, the KO AM gave a markedly different picture with 2 proteins that were significantly higher in females and only 1 in males. As in the protease balance/chaperone function group nearly half (13 of 28) of the significant changes relative to KO values were in the 18 hr time point (Additional file 5 – Table B).

However, in the actin-related/cytoskeletal protein group there were fewer significant changes between KO, WT, and the treatment groups in the female mice (n = 17)(Additional file 4 – Table B) than in the male mice (n = 32)[29] suggesting a more SP-A-responsive actin-related protein group in the males

c) *Nrf2-related protein group:* Sex differences were also apparent in the Nrf2-regulated proteins (Additional file 5 – Table C). Although in WT there were only 3 significant differences (2 higher in females; 1 in males) between males and females, at 18 hr after SP-A treatment there were 8 (5 higher in females; 3 in males).

The Nrf2-related proteins also had very different sex-specific response patterns with a total of 7 significant differences in the female (Additional file 4 – Table C), but 17 [29] in the male.

d) *Other groups:* There were relatively few (n = 2) sex differences in the regulatory/differentiative proteins (Additional file 5 – Table D). This group also had very few significant differences when KO were compared to the WT and SP-A treatment groups in both females (Additional file 4 – Table D) and males [29]. In the regulation of inflammation proteins (Additional file 5 – Table E) more sex differences were apparent and more than half of these were found in mice treated for 18 hr with SP-A. Comparing the SP-A response patterns showed the females to have slightly fewer (n = 10) significant changes versus KO than in the males (n = 14) [29].

#### Changes in proteins involved in estrogen action

Some of the identified proteins have known roles in mediating estrogen activity. These include major vault protein [105], chaperonin subunit 2-(beta) [106], alpha enolase [107], Rho GDP dissociation inhibitor alpha [108], and heat shock protein 1-beta (HSP90AB1) [109]. Several of these proteins were expressed at significantly higher levels in female WT mice than in male WT (major vault protein, chaperonin subunit 2-beta, and rho GDP dissociation inhibitor alpha). Alpha enolase and heat shock protein 1-beta were not significantly different.

## Discussion

Male WT C57BL/6 mice in response to *K. pneumoniae* infection exhibit a higher level of bacterial dissemination [23], more pronounced extrapulmonary lesions in liver and spleen [22], and lower survival rates than their female counterparts [20]. However, when an oxidative stress, in the form of acute ozone exposure, is imposed prior to infection, the survival advantage shifts to the males and they survive at higher rates than the females [20] who develop a more excessive lung inflammatory response compared to males [22]. We hypothesized that SP-A and circulating sex hormones are responsible for these sex differences. Our recent studies indicate a role of sex hormones in the observed sex differences [24]. Because of the pivotal role of the AM in alveolar host defense, in this study, we used 2D-DIGE to examine the interaction of sex and SP-A on the proteome of the AM. The role of SP-A was studied by treating SP-A KO mice with exogenous SP-A for 6 and 18 hr and studying the resulting effects on the AM proteome. We have previously reported the results obtained when AM from male mice were analyzed [29]. Here we analyzed the results in AM from females and compared them with the males.

The most striking aspect of this study was the fact that the effect of SP-A had very different characteristics in females than in males. This manifested itself in several ways. When the total numbers of significantly changing protein spots and identified whole proteins were examined, in females there were many fewer changes in the 18 hr SP-A treatment and WT groups when each was compared to KO as opposed to what we observed for the males (Figure 2 and Table 2 versus our previously published study in males (see Additional file 2 in [29])). Furthermore, the principal component analysis that compared male and female WT and KO mice showed a much greater separation between the male and female WT groups than the KO groups, indicating that SP-A may play a role in the observed sex differences.

Our previous study of male AM showed that many proteins are lower in AM [29] from WT mice than in KO. These findings could reflect either an SP-A-mediated down-regulation of several proteins or a compensation for the absence of SP-A. In either case, many proteins are increased in KO mice. However, this trend is less pronounced in AM from female mice (Table 1) and there are only about half as many significant differences (in all comparisons to KO) as there are in male AM. Direct comparison of males [29] and females (present study) in response to SP-A revealed sex differences. There were equal numbers of proteins at their highest levels in WT male and female, but in KO mice (in the absence of SP-A) ~70% of the changing proteins were higher in the males, although few of these reached statistical significance. This may indicate that while SP-A plays an important role in AM protein expression, its effect is more pronounced in the male and somewhat more limited in the female. This scenario is consistent with reports showing an inhibitory effect of estrogen on inflammatory processes in lung and other systems [110,111].

The most responsive group of proteins in response to SP-A was that related to actin function, which consisted of 38 proteins and underwent the most changes. However, the SP-A-induced changes were very similar in males and females, although there were more significant differences in males. Furthermore, SP-A response patterns in proteins related to regulation of inflammation (ROI) and regulatory/developmental function (RDP) were similar in both sexes. However, in two cases we observed marked differences in the response patterns between sexes – especially in proteins involved in protease balance/chaperone function (PBCF) and to a slightly lesser degree in Nrf2-regulated proteins (NRF) (see Additional file 4 Table A and Table C). In the females there were only about half as many significant changes in the WT and SP-A treated groups relative to KO as in the males. However we observed an interesting trend in these two functional groups. In both groups (PBCF and NRF), the relative numbers of proteins expressed at decreased levels in WT relative to KO were comparable in both sexes, but when mice were treated with SP-A, the pattern shifted substantially in females and much less so in males. Indeed, within 6 hr of SP-A treatment, while males [29] continued to express most of the proteins in each group at lower levels than in KO, in the females many more proteins were expressed at higher levels than in KO. This trend was moderated somewhat by 18 hr after SP-A treatment, but although the relative numbers of increased and decreased proteins versus KO in these two functional groups were almost identical to the WT/KO comparison in males, they were still quite different in females. These sex differences may be the basis for differential responses and sensitivity to oxidative stress and its consequences between the sexes [5,112-114].

Although there seems to be an interaction between sex and the effect of SP-A, it is unclear at this point what the responsible mechanisms are. However, there were several proteins that were expressed at significantly higher levels in females than in males in WT or in KO mice that are known to interact with the estrogen receptor and may play a role in this interaction. These include the major vault protein [105], chaperonin subunit 2 (beta) (CCT2), Rho GDP dissociation inhibitor alpha [108], and heat shock protein 1 beta. The major vault protein is induced by a variety of cellular stresses [115] and has been implicated in defense against bacterial infection [116]. We speculate that its higher levels and estrogen dependence in the female [105] contributes to the survival advantage we previously reported in females infected with *K. pneumoniae*[21].

If the changes in protein expression and SP-A responsiveness are indeed due to the influence of circulating estrogen, as is the case with the female survival advantage against *K. pneumoniae* infection [24], it is possible that these changes in expression may vary somewhat with the alterations in estrogen levels accompanying the various stages of the estrous cycle. However, since cycle stage was not determined in this study it is not possible to determine whether the effects reported here are estrogen dose-dependent or whether they are just due to the presence of estrogen in the circulation.

A number of studies have compared inflammatory mechanisms in males and females and evidence continues to increase that differences in these mechanisms are the basis for sex-specific differences in the severity and clinical course of lung diseases with an inflammatory component. Estrogens have been described as having a more efficient and protective role in an animal model of lung inflammation [117], as well as in other inflammatory contexts [118], and have been characterized as important pleiotropic regulators of inflammatory function [119,120]. We postulate that a dynamic interaction between SP-A and estrogen in terms of regulating chaperones, proteases, and proteins regulated by Nrf2 in the lung may be responsible for these sex differences.

## Conclusions

The AM proteome is markedly different in KO mice than in WT mice, but rescue with exogenous SP-A restores many of the characteristics of the WT AM proteome to KO mice not only as shown in males [29], but also in females (present study). However, although the magnitude and extent of the SP-A-related changes seems to be more pronounced in male mice, with the expression of many more proteins being significantly altered in response to SP-A, the females undergo very pronounced changes in the expression of two protein groups (protease balance/chaperone function and Nrf2-related) that appear to be more affected in the females than in the males.

## Materials and methods

### Animals

This study was done using pathogen-free WT and SP-A KO female mice on the C57BL/6 genetic background. WT mice were obtained from Jackson Laboratories (Bar Harbor, ME). Breeder pairs of SP-A KO mice had been obtained from Dr. Samuel Hawgood at the University of California, San Francisco and were propagated and raised under specific pathogen-free conditions in a barrier facility at the Penn State College of Medicine [121]. The SP-A KO mice and sentinel mice housed in the same room showed no evidence of respiratory pathogens. The Institutional Animal Care and Use Committee at the Penn State College of Medicine approved this study.

This study used a total of 16, 25–34 g C57BL/6 WT and SP-A KO female mice. These were divided into four groups with 4 animals per group: 1) SP-A KO control (baseline) mice that did not receive any treatment; 2) SP-A KO mice that were treated with SP-A and sacrificed 6 hr after treatment; 3) SP-A KO mice that were treated with SP-A and sacrificed 18 hr after SP-A treatment; and 4) WT control (baseline) mice that did not receive any treatment. The female mice used in this study were compared to male mice that had undergone identical manipulations and described in detail previously [29].

### SP-A preparation

SP-A was purified from the BAL fluid from normal human lungs obtained from organ donors. The protocol was approved by the Penn State College of Medicine Institutional Review Board. Donor lungs were lavaged with 0.9% saline and the lavage fluid collected and centrifuged at 150 × g for 10 min at 4°C to obtain cell-free BAL. SP-A was then purified by repeated precipitation with 5 mM calcium chloride after which its purity was checked by 1D-PAGE with silver stain and by Western blot and determined to be greater than 99 percent pure. We also performed an LPS determination with the QCL-1000 Limulus Amebocyte Lysate assay (Lonza, Walkersville, MD) and found the LPS content of a 1 μg sample of SP-A to be below the detectable limit of the assay (0.1 EU/ml) or < 500 fg of LPS/μg of SP-A.

### Treatment of mice with SP-A and collection of alveolar macrophages

Experimental manipulations and harvesting of samples has been described in detail previously [29]. Briefly, the mice were anesthetized with Ketamine HCl (Ketaject, Phoenix Pharmaceuticals Inc., St. Joseph, MO) and Xylazine (XYLA-JECT, Phoenix Pharmaceuticals Inc., St. Joseph, MO) and SP-A was instilled intrapharyngeally with 5 μg of normal human SP-A in 50 μL of 0.9% sodium chloride containing 2 mM calcium chloride. The 5 μg dose of SP-A/mouse was based on our SP-A determinations of total BAL SP-A from C57BL/6 mice (mean 3.2 μg; n = 9) and a previous study that found this dose to be sufficient to produce tubular myelin in the BAL of SP-A KO mice [122].

To obtain AM mice were anesthetized and subjected to BAL at intervals of 6 hr and 18 hr following treatment with SP-A. Six hours was chosen as our initial time point because a previous study found tubular myelin at that time [122]. Furthermore, we postulated that this time interval would be sufficient for new protein synthesis to occur in response to SP-A treatment. The 18 hr time point was chosen to determine the longer term effects of a single dose of SP-A, that could potentially include the consequences of the indirect effects of SP-A. AM were obtained by performing BAL with PBS, 1 mM EDTA using a volume equal to 80% of lung vital capacity (5 times with 0.5 mL) for a total of 2.5 ml. The fluid was instilled and withdrawn 3 times with chest massage during withdrawal, then centrifuged at 150 × g for 5 min at 4°C and the cell pellet washed with 1 mL of PBS, 1 mM EDTA. Cells were counted to obtain total and differential cell counts before being frozen at −80°C for subsequent proteomic studies.

To prepare AM for 2D-DIGE frozen AM pellets were lyophilized until completely dry and resuspended in 25 μL of standard cell lysis buffer (30 mM Tris–HCl, 2 M thiourea, 7 M urea, 4% CHAPS, pH 8.5). Protein determinations were done using the Bio-Rad Protein Assay (Bio-Rad, Hercules, CA) and the concentration of protein was adjusted to 1 mg/ml for CyDye labeling.

### CyDye labeling (minimal labeling) and electrophoresis for 2D-DIGE

These procedures have been described in detail previously [29,30,123]. Information about the 2D-DIGE study is provided in a form that complies with the most recent version <http://www.psidev.info/miape/MIAPE_GE_1_4.pdf> of Minimum Information About a Proteomics Experiment – Gel Electrophoresis (MIAPE-GE) standards currently under development by the Human Proteome Organization Proteomics Standards Initiative (see Additional file 6).

### Gel imaging, image analysis, and statistics

Information about the acquisition and processing of data from the 2D-DIGE studies are provided in the form that complies with the most recent version of the guidelines established for Minimum Information about a Proteomics Experiment – Gel Informatics (MIAPE-GI) currently under development by the Human Proteome Organization Proteomics Standards Initiative http://www.psidev.info/files/miape-gi-v1.pdf (see Additional file 7). Gel images were imported into the Progenesis SameSpots v4.0 program (Nonlinear Dynamics) for analysis. For identified proteins having multiple isoforms, the normalized volumes of all isoforms of a given protein were added together and statistical analysis was performed on the totals using Microsoft Excel.

### Protein identification by mass spectrometry

We have used this procedure in previous studies for other types of protein samples [28,30,123] and we recently published a detailed account including many modifications and refinements [29].

All 791 gel spots were picked robotically and processed for analysis by MALDI-ToF/ToF mass spectrometry (5800 Proteomic Analyzer Applied Biosystems, Foster City, CA) in the Mass Spectrometry Core at the Penn State University College of Medicine. The MS and MS/MS data were submitted to the MASCOT search engine using the NCBI non-redundant database and mouse taxonomy for identification. The search parameters included: trypsin digestion with a maximum of three missed cleavages; fixed modifications, carbamidomethylation; variable modifications, carbamylation, acetylation, deamidation, oxidation; peptide mass tolerance, 0.15 Da. MASCOT confidence interval scores of > 95% combined with a ProteinPilot score of greater than 61 were considered as a positive protein identification. An image of the reference gel is shown in Additional file 2 with all identified proteins circled and numbered. From the 791 gel spots analyzed, 290 spots were identified resulting in 76 unique proteins.

The PANTHER database and the scientific literature were used to provisionally assign molecular function and biological process to each identified protein. We then re-assigned the identified proteins to five broad functional classes as we described previously [29] including: a) actin-related/cytoskeletal proteins; b) proteins involved in protease balance/chaperone function; c) proteins involved in regulation of inflammation; d) proteins involved in regulatory/differentiative processes; and e) proteins regulated by Nrf2. It should be noted that some proteins are in more than one functional group. This classification scheme was more inclusive than relying solely on the biological function classification provided by PANTHER and similar gene ontology databases. We also used the Ingenuity Pathway Analysis program (Ingenuity Systems, Redwood City, CA) to gain additional insight into the functional significance of the observed changes. Protein names, accession numbers, and the functional groups we assigned them to are listed in Additional file 1 together with a list of supporting references.

## Abbreviations

2D-DIGE: Two-dimensional difference gel electrophoresis; AM: Alveolar macrophages; KO: SP-A knockout mice; MALDI-ToF/ToF: Matrix-assisted laser desorption ionization-time of flight/time of flight; SP-A: Surfactant protein A; WT: Wild type mice.

## Competing interests

The authors have no competing interests.

## Authors’ contributions

DSP and JF designed the study. DSP interpreted data and prepared the manuscript. JF assisted with data interpretation, and participated in manuscript preparation. TMU treated mice, collected samples, ran gels, prepared mass spec samples, evaluated mass spec data, did preliminary analysis of proteomic data, and participated in the writing of the manuscript. All authors read and approved the final manuscript.

## Supplementary Material

Additional file 1 **File format: doc.** Title: Protein names and cross references to accession numbers and categories. Description: File containing a table that has the gel numbers and names of all identified proteins. It contains both NCBI GI numbers and Swiss Prot Accession numbers for all proteins, as well as the designation for the functional group(s) to which each protein was assigned. The citations used to assign each protein to the designated functional category are part of the reference list for the main article. Click here for file

Additional file 2 **Reference gel (same as in male study).** Description: Spot map showing location of all identified proteins. Click here for file

Additional file 3 **Title: Values for all identified female alveolar macrophage proteins with note of significant changes.** Description: File containing a table that gives normalized volumes for all proteins for each individual group +/- SD and indicates comparisons between groups that were significantly different. Click here for file

Additional file 4 **Tables A – F.** Tables for each protein functional group (Tables A-E) and for all proteins (Table F) summarizing changes of each female group relative to KO baseline values. The data for each functional group (Tables A-E) are extracted from the complete list presented in Table F. Click here for file

Additional file 5 **Tables A – F.** Tables for each protein functional group (Tables A-E) and for all proteins (Table F) summarizing sex differences for each treatment group. The data for each functional group (Tables A-E) are extracted from the complete list presented in Table F. Click here for file

Additional file 6 **Title: MIAPE: Gel Electrophoresis.** Description: File containing Minimum Information About a Proteomics Experiment – Gel Electrophoresis in the format recommended by the Human Proteome Organization Proteomic Standards Initiative. Click here for file

Additional file 7 **Title: MIAPE: Gel Informatics.** Description: File containing Minimum Information About a Proteomics Experiment – Gel Informatics in the format recommended by the Human Proteome Organization Proteomic Standards Initiative. Click here for file
