# Supplementary material for: Sex differences in the response of the alveolar macrophage proteome to treatment with exogenous surfactant protein-A
Source: Proteome Sci. 2012 Jul 23;10:44. doi: 10.1186/1477-5956-10-44 (PMC3570446; doi:10.1186/1477-5956-10-44)
Supplement: Additional file 1 — File format: doc. Title: Protein names and cross references to accession numbers and categories. Description: File containing a table that has the gel numbers and names of all identified proteins. It contains both NCBI GI numbers and Swiss Prot Accession numbers for all proteins, as well as the designation for the functional group(s) to which each protein was assigned. The citations used to assign each protein to the designated functional category are part of the reference list for the main article. [file 1477-5956-10-44-S1.doc]

Additional File 1

MIAPE: Gel Electrophoresis

Version 1.4, January, 2008

Reporting requirements for gel electrophoresis

1. General features

1.1.1 Date stamp: 2010-06-09

1.1.2 Responsible person or institutional role: Todd M. Umstead, Senior Research Support Associate, Penn State Center for Host defense, Inflammation, and Lung Disease (CHILD) Research, Department of Pediatrics, P.O. Box 850, Hershey, PA 17033

1.1.3 Electrophoresis type: 2D-DIGE with PAGE electrophoresis

2. Sample

2.1.1 Sample name(s): Cy-labeled Mouse alveolar macrophage (mAM) proteins from baseline untreated SP-A (-/-) (knockout) male mice (KOM), baseline untreated SP-A (-/-) (knockout) female mice (KOF), baseline untreated wild-type male mice (WTM), baseline untreated wild-type female mice (WTF), SP-A (-/-) male mice 6 hours after treatment with SP-A (KOM 6hr SP-A), SP-A (-/-) female mice 6 hours after treatment with SP-A (KOF 6hr SP-A), SP-A (-/-) male mice 18 hours after treatment with SP-A (KOM 18hr SP-A), and SP-A (-/-) female mice 18 hours after treatment with SP-A (KOF 18hr SP-A). Four replicates from each group (KOM, KOF, WTM, WTF, KOM 6hr SP-A, KOF 6hr SP-A, KOM 18hr SP-A, KOF 18hr SP-A) Cy3/Cy5 counterbalanced to eliminate dye-based artefacts

2.1.2 Loading buffer (analytical gels): Samples were resuspended and labeled in GE Healthcare (GE) standard cell lysis buffer (option 1) and equal amounts (20μg) of Cy3-labeled sample, Cy5-labeled sample, and Cy2-labeled pool samples were combined for each gel (60μg total), and an equal volume of GE 2X sample buffer containing 2% IPG buffer (pH 4-7) added to bring the final volume to 140μL

2.1.3 Loading buffer (preparative/picking gels): Equal amounts of each sample were pooled (450 μg total combined) and resuspended in GE Healthcare (GE) standard cell lysis buffer (option 1), an equal volume of GE 2X sample buffer containing 2% IPG buffer (pH 4-7) and 1.2% DeStreak reagent was added to each sample, samples were brought up to a final volume of 450μL with GE DeStreak^TM^ Rehydration Solution containing 0.5% IPG buffer (pH 4-7)

3. Gel matrix and electrophoresis

***First-dimension electrophoresis***

3.1. Dimension details

3.1.1 Ordinal number for this dimension: First

3.1.2 Separation method employed: Isoelectric focusing (IEF)

3.2 Gel matrix

3.2.1 Description of gel matrix: Immobiline DryStrip, 24 cm (pH 4-7)

3.2.2 Gel manufacturer: GE Healthcare, part numbers 17-6002-46 (pH 4-7) - (strip ID numbers for pH 4-7 analytical gels: 56463, 56464, 56589, 56590, 56591, 56592, 56593, 56594, 56595, 56597, 56598, 56599, 73401, 73406, 73410, 73411, 73412, 73419, 73420, 73421, 73422, 73423, 73424, 73425) (strip number for pH 4-7 picking gel: 99068)

3.2.3 Physical dimensions: Cartesian Coordinates of 235mm-L x 3mm-W x 0.5mm-D

3.2.4 The physicochemical property range and distribution (as appropriate): pH 4-7

3.2.5 Acrylamide concentration: N/A

3.2.6 Acrylamide : Crosslinker ratio: N/A

3.2.7 Additional substances in gel: N/A

3.2.8 Gel lane: N/A

3.2.9 Sample application (analytical gels): Samples from each group were randomly assigned to Cy3 or Cy5 to ensure no dye-based artefacts in quantitation. Aliquots of 20 μg of mAM protein from each sample were labeled with Cy3 or Cy5 (200 picomoles). A normalization pool was created by combining equal amounts of protein from every sample and an aliquot of the pool was labeled with Cy2 (200 picomoles). Equal amounts (20μg) of Cy3-labeled sample, Cy5-labeled sample, and Cy2-labeled pool samples were mixed in buffer as described in 2.1.2 and applied to IPG strips (prehydrated with 450 μL Destreak^TM^ Rehydration solution [GE]) using cup loading and the IPGphor Cup Loading Manifold

3.2.10 Sample application (preparative/picking gels): Equal amounts of each sample were pooled (450 μg total combined) and resuspended in buffer as described in 2.1.3 and applied to IPG strips using in-strip rehydration

3.3 Protocol

3.3.1 Buffers: See 2.1.2 and 2.1.3 above, strips rehydrated and run under GE PlusOne DryStrip strip cover fluid following GE Ettan DIGE System User Manual (18-1173-17 AB)

3.3.2 Electrophoresis conditions (analytical gels): Isoelectric focusing was done using an IPGphor 3 apparatus (GE) at 20°C under DryStrip cover fluid with the following voltages and times: 3 hour at 300 V (step and hold); 7 hour at 1000 V (gradient); 4 hour at 8000 V (gradient); 4 hour at 8000 V (step and hold)

3.3.3 Electrophoresis conditions (preparative/picking gels): Isoelectric focusing was done using an IPGphor 3 apparatus (GE) at 20°C under DryStrip cover fluid with the following voltages and times: 15 hour at 0 V (passive rehydration); 6 hour at 30 V (active rehydration); 3 hour at 300 V (step and hold); 3 hour at 600 V (gradient); 3 hour at 1000 V (gradient); 3 hour at 8000 V (gradient); 4 hour at 8000 V (step and hold)

***Second-dimension electrophoresis***

3.1. Dimension details

3.1.1 Ordinal number for this dimension: Second

3.1.2 Separation method employed: Sodium dodecyl sulphate polyacrylamide gel electrophoresis (SDS-PAGE)

3.2 Gel matrix

3.2.1 Description of gel matrix: Ettan DALT 10% polyacrylamide slab gel, reducing SDS

3.2.2 Gel manufacturer: Gels manufactured ‘in-house’ using the Ettan DALTtwelve Gel Caster (GE) and following the GE Ettan DALTtwelve System User Manual (80-6476-53/Rev.AC/10-02)

3.2.3 Physical dimensions: Cartesian Coordinates of 20.5cm-L x 25.5cm-W x 1mm-D

3.2.4 The physicochemical property range and distribution: logarithmic apparent molecular mass 200-10 kDa

3.2.5 Acrylamide concentration: 10% polyacrylamide resolving gel with no stacking gel

3.2.6 Acrylamide : Crosslinker (N,N’-methylene-bis-acrylamide) ratio: 29:1 (3.3% C) (Bio-Rad Laboratories Electrophoresis Purity Reagent, Catalog #161-0156)

3.2.7 Additional substances in gel: All details of substances and reagents can be found in the GE Ettan DALTtwelve System User Manual (80-6476-53/Rev.AC/10-02).

3.2.8 Gel lane: N/A

3.2.9 Sample application: A single Immobiline DryStrip from the above described first-dimension was applied to the top of each Ettan DALT gel and sealed using Bio-Rad ReadyPrep Overlay Agarose (0.5% in 1X TGS with BFB) (Cat# 163-2111)

3.3 Protocol

3.3.1 Buffers: Running buffers were used as described in the GE Ettan DALTtwelve System User Manual (80-6476-53/Rev.AC/10-02) with 7.5L of 1X Tris/Glycine/SDS buffer in the lower (anodal) buffer chamber and 2.5L of 2X Tris/Glycine/SDS buffer in the upper (cathodal) buffer chamber. Both the 1X and 2X running buffers were prepared using Bio-Rad 10X Tris/Glycine/SDS Buffer for SDS PAGE applications (Catalog #161-0732)

3.3.2 Electrophoresis conditions: 5W per gel for 30 min at 20°C then 15W per gel at 20°C until the bromophenol blue dye front was ½ cm from the bottom of the gel (~4 hr) using the GE Ettan DALTtwelve System Separation Unit

4. Inter-dimension process (not applicable for one-dimensional gel electrophoresis)

4.1 Inter-dimension process

4.1.1 Step name: Equilibration of IPG strips after first-dimension/reduction and alkylation

4.1.2 Inter-Dimension buffer: Equilibration solution (50mM TrisCl, 6M urea, 30% glycerol, 2% sodium dodecyl sulphate

4.1.3 Additional reagents: 0.5% dithiothreitol (DTT) in first equilibration solution and 4.5% iodoacetamide in second equilibration solution

4.1.4 Equipment: N/A

4.1.5 Protocol: IPG strips were equilibrated for 15 minutes with shaking in equilibration solution containing DTT and then for 15 minutes with shaking in equilibration solution containing iodoacetamide as described in 4.1.3 above and in the GE Ettan DALTtwelve System User Manual (80-6476-53/Rev.AC/10-02)

5. Detection (if applicable)

5.1 Direct detection

5.1.1 Name of direct detection process: Detection of proteins on analytical gels was done by labeling proteins prior to electrophoresis with spectrally resolvable CyDye DIGE Fluor minimal dyes (Cy2, Cy3 and Cy5) (GE) that are matched for mass and charge. Preparative/picking gels were post-stained using Deep Purple Total Protein stain (GE catalog #RPN6305)

5.1.2 Direct detection agents: See 5.1.1 above

5.1.3 Additional reagents and buffers: Additional reagents and buffers for CyDye DIGE Fluor minimal dyes can be found in the GE Ettan DALTtwelve System User Manual (80-6476-53/Rev.AC/10-02). Additional reagents and buffers for Deep Purple Total Protein gel stain included: Fix solution (30% ethanol, 7.5% acetic acid), Deep Purple solution (1:200 Deep Purple reagent in 100mM sodium borate [pH 10.5]), Wash solution (15% ethanol), and Acidification solution (15% ethanol, 1% citric acid [pH 2.3]).

5.1.4 Equipment: Images were collected using a Typhoon 9410 Scanner (GE) (see 6. Image acquisition below)

5.1.5 Direct detection protocol: Protocol for CyDye DIGE Fluor minimal dyes can be found in the GE Ettan DALTtwelve System User Manual (80-6476-53/Rev.AC/10-02). Protocol for use of Deep Purple Total Protein stain included: Fixing overnight with 30% ethanol, 7.5% glacial acetic acid, staining with Deep Purple solution for 2 hr, washing for 30 min, and acidification for 30 min

5.2 Indirect detection

5.2.1 Name of indirect detection process: N/A

5.2.2 Transfer medium: N/A

5.2.3 Detection medium: N/A

5.2.4 Indirect detection agents: N/A

5.2.5 Additional reagents and buffers: N/A

5.2.6 Equipment: N/A

5.2.7 Indirect detection protocol: N/A

6. Image acquisition (if applicable)

6.1 Acquisition equipment

6.1.1 Type of equipment: Variable-mode imager that produces digital images of radioactive, fluorescent, or chemiluminescent samples

6.1.2 Name of equipment: Typhoon 9410 Variable Mode Imager (GE)

6.1.3 Software: ImageQuant TL (GE)

6.1.4 Calibration (if appropriate): Automatic calibration

6.1.5 Equipment specific parameters: All gels were imaged in fluorescence mode using the DIGE Ettan DALT sample tray area with a focal plane of 3mm at a resolution of 100μm. Photomultiplier tube voltages were individually set for each of the three colored lasers to ensure maximum, linear signals. The same voltages were used for all gels. DIGE Gels were imaged at three different wavelengths (Cy2: Blue2 [488] laser – Em 520nm BP 40, Cy3: Green [532] laser – Em 580nm BP 30, Cy5: Red [633] laser – Em 670nm BP 30) and the Deep Purple stained gels were imaged with a separate filter (Green [532] laser – Em 610nm BP 30)

6.2 Acquisition protocol

6.2.1 Image acquisition process: See 6.1.5 above

6.2.2 Reference to gel matrix: Ettan DALT 10% polyacrylamide slab gel (see also section 3 and 6.1.5 above)

7. Image (as a result of section 6)

7.1.1 Image name (or id): Images were named according to the ID number of the Immobiline DryStrip from the first-dimension gel (see 3.2.2 above), PMT voltage setting, date of first dimension run, and the dye that was used to stain the sample (ie. Cy2 Standard, Cy3, Cy5, or Deep Purple Picking) and have a GEL image format (.gel)

7.1.2 Dimensions: 2800 pixels x 2200 pixels

7.1.3 Resolution: 100 micrometers per pixel (μm/pixel)

7.1.4 Bit depth: 16 bit

7.1.5 Image location: Images available upon request

7.1.6 Standard image orientation: Lowest pH value on the left with highest on the right for first-dimension IEF, and high molecular weight proteins at the top with low at the bottom for second-dimension SDS-PAGE
