# Supplementary material for: Sex differences in the response of the alveolar macrophage proteome to treatment with exogenous surfactant protein-A
Source: Proteome Sci. 2012 Jul 23;10:44. doi: 10.1186/1477-5956-10-44 (PMC3570446; doi:10.1186/1477-5956-10-44)
Supplement: Additional file 3 — Title: Values for all identified female alveolar macrophage proteins with note of significant changes. Description: File containing a table that gives normalized volumes for all proteins for each individual group +/- SD and indicates comparisons between groups that were significantly different. [file 1477-5956-10-44-S3.doc]

**Additional File 3**

**Protein names and cross references to accession numbers and categories**.

| **Gel No.** | **Protein Name** | **NCBI**  **GI Number** | **Swiss-Prot**  **Access. No.** | **Functional**  **Categories** | **Refs.** |
| --- | --- | --- | --- | --- | --- |
| 1 | 65-kDa macrophage protein | gi|984636 | Q61233 | ARC,NRF | [31,32] |
| 2 | Actin related protein 2/3 complex, subunit 5 | gi|224809382 | Q9CPW4 | ARC | [33,34] |
| 3 | Actin-related protein 3 | gi|12835802 | Q99JY9 | ARC | [33] |
| 4 | Actr2 protein | gi|29126784 | P61161 | ARC | [33] |
| 5 | Alpha-fetoprotein | gi|191765 | P07724 | ROI | [35] |
| 6 | Annexin A2 | gi|6996913 | P07356 | ARC, ROI | [36-38] |
| 7 | Annexin A4 | gi|33416530 | Q7TMN7 | ROI | [39] |
| 8 | Anxa 5 protein | gi|13277612 | P48036 |  |  |
| 9 | ArsA arsenite transporter, ATP-binding, homolog 1 | gi|12025542 | O54984 |  |  |
| 10 | Atp5b protein | gi|23272966 | P56480 |  |  |
| 11 | Calpain, small subunit 1 | gi|110227381 | O88456 | ARC, PBCF | [40,41] |
| 12 | Capping protein (actin filament) muscle Z-line, alpha 2 (CapZ alpha-2) | gi|6671672 | P47754 | ARC | [34,42] |
| 13 | Capping protein (actin filament) muscle Z-line, beta isoform (CapZ beta) | gi|83649737 | P47757 | ARC | [34,42] |
| 14 | Cathepsin D precursor | gi|6753556 | P18242 | PBCF, NRF | [43,44] |
| 15 | Chaperonin subunit 2 (beta) (CCT2) | gi|126521835 | Q542X7 | ARC,PBCF | [45,46] |
| 16 | Chia protein | gi|15029822 | Q91XA9 | ROI | [47] |
| 17 | Chitinase 3-like 3 precursor (Ym1) | gi|254281348 | O35744 | ROI | [47] |
| 18 | Chitinase-related protein MCRP | gi|1336166 | Q61201 | ROI | [48] |
| 19 | Chloride intracellular channel 1 | gi|15617203 | Q9Z1Q5 | ARC, PBCF | [49,50] |
| 20 | Chloride intracellular channel 4 (mitochondrial) | gi|7304963 | Q9QYB1 | ARC, NRF | [51,52] |
| 21 | CNDP dipeptidase 2 | gi|31981273 | Q9D1A2 | PBCF | [53] |
| 22 | Coactosin-like 1 | gi|19482160 | Q9CQI6 | ARC, PBCF | [54,55] |
| 23 | EF hand domain containing 2 | gi|31981086 | Q8C845 |  |  |
| 24 | Eno1 protein (Alpha-enolase) | gi|34784434 | Q6PHC1 | ARC, PBCF, ROI | [39,56,57] |
| 25 | Eukaryotic translation initiation factor 5A | gi|56800106 | P63242 | ARC, RDP, ROI | [58] |
| 26 | Ezrin | gi|50881 | P26040 | ARC | [40] |
| 27 | F-actin capping protein alpha-1 subunit (CapZ alpha-1) | gi|161086971 | P47753 | ARC | [34,42] |
| 28 | Ferritin heavy chain 1 | gi|6753912 | P09528 | NRF | [32] |
| 29 | Ferritin light chain 1 | gi|114326466 | Q9CPX4 | NRF | [32] |
| 30 | Gamma-actin | gi|809561 | P63260 | ARC, NRF | [59,60] |
| 31 | Gelsolin precursor | gi|28916693 | P13020 | ARC, NRF | [59,60] |
| 32 | Glucose-6-phosphate dehydrogenase X-linked | gi|6996917 | Q00612 | NRF | [61] |
| 33 | Guanine deaminase | gi|6753960 | Q9R111 | ARC | [62] |
| 34 | Heat shock protein 1, beta (HSP90AB1) | gi|40556608 | Q71LX8 | ARC, NRF, PBCF, ROI | [60,63,64] |
| 35 | Heat shock protein 5 precursor (GRP78) | gi|254540166 | P20029 | NRF, PBCF, ROI | [32,63,65,66] |
| 36 | Heat shock protein 65 (HSP60) | gi|51455 | P63038 | PBCF, ROI | [63,66,67] |
| 37 | Heat shock protein 8 (HSC70; HSC71) | gi|42542422 | P63017 | PBCF, ROI | [63,66] |
| 38 | Heat shock protein 90, beta (Grp94), member 1 | gi|14714615 | Q91V38 | NRF, PBCF, ROI | [63,66] |
| 39 | Hematopoietic cell specific Lyn substrate 1 | gi|255760028 | Q922I8 | ARC, ROI | [68,69] |
| 40 | Heme-binding protein | gi|3724328 | Q9R257 | ROI | [70] |
| 41 | Heterogeneous nuclear ribonucleoprotein K | gi|13384620 | P61979 | RDP | [71] |
| 42 | High mobility group 1 protein | gi|600761 | P63158 | RDP, ROI | [72] |
| 43 | Hnrpf protein | gi|58476100 | Q9Z2X1 | RDP | [73] |
| 44 | Kappa-B motif-binding phosphoprotein | gi|1083569 | Q9YH06 | RDP | [74] |
| 45 | Keratin complex 2, basic, gene 8 | gi|114145561 | P11679 | ARC, NRF | [32,75,76] |
| 46 | Keratin type II | gi|511654 | P11679 | ARC | [76] |
| 47 | Krt13 protein | gi|37994713 | P08730 | ARC, NRF | [32,76,77] |
| 48 | Laminin receptor | gi|293694 | P14206 |  |  |
| 49 | Major vault protein (MVP) | gi|17433104 | Q9EQK5 | ARC | [78] |
| 50 | Microtubule-associated protein, RP/EB family, member 1 | gi|7106301 | Q61166 | ARC | [79] |
| 51 | Myosin light chain, regulatory B-like | gi|71037403 | Q6ZWQ9 | ARC | [59] |
| 52 | Nucleophosmin 1 | gi|55153941 | Q5U438 | RDP | [80] |
| 53 | p50b, Leukocyte-specific protein 1 (LSP1) | gi|728498 | P19973 | ARC, RDP, ROI | [81,82] |
| 54 | Peroxiredoxin 2 | gi|148747558 | Q61171 | NRF | [83] |
| 55 | Prolyl 4-hydroxylase, beta polypeptide precursor | gi|42415475 | Q922C8 | PBCF | [84] |
| 56 | Proteasome (prosome, macropain) 28 subunit, alpha | gi|6755212 | P97371 | PBCF | [85] |
| 57 | Proteasome alpha 1 subunit | gi|33563282 | Q9R1P4 | PBCF, NRF | [86,87] |
| 58 | Protein disulfide isomerase associated 6 (PDI-P5) | gi|60502437 | Q922R8 | PBCF | [88] |
| 59 | Protein disulfide-isomerase A3 precursor | gi|112293264 | P27773 | PBCF | [89] |
| 60 | Protein synthesis initiation factor 4A | gi|556308 | P60843 | NRF, RDP | [32,90] |
| 61 | Purine nucleoside phosphorylase | gi|388921 | P23492 |  |  |
| 62 | Put. beta-actin (aa 27-375) | gi|49868 | P60710 | ARC, NRF | [59,91] |
| 63 | Rab GDP dissociation inhibitor beta | gi|26348171 | Q61598 | ARC | [92] |
| 64 | Rho GDP dissociation inhibitor (GDI) alpha | gi|31982030 | Q99PT1 | ARC | [93] |
| 65 | Rho, GDP dissociation inhibitor (GDI) beta | gi|33563236 | Q61599 | ARC | [93] |
| 66 | Serine (or cysteine) proteinase inhibitor, clade B, member 1a | gi|114158675 | Q9D154 | PBCF | [94] |
| 67 | Stathmin | gi|14625464 | Q91XT3 | ARC | [95] |
| 68 | Superoxide dismutase 1, soluble | gi|45597447 | P08228 | NRF | [32] |
| 69 | Tropomodulin 3 | gi|8394460 | Q9JHJ0 | ARC | [42] |
| 70 | Tropomyosin 3, gamma | gi|40254525 | Q8K0Z5 | ARC | [42] |
| 71 | Tubulin, beta 5 | gi|7106439 | P99024 | ARC, NRF | [60,96] |
| 72 | Tyrosine 3/tryptophan 5 -monooxygenase activation protein, ε (14-3-3ε) | gi|5803225 | P62258 | ROI | [97] |
| 73 | Tyrosine 3-monooxyg./tryptophan 5-monooxyg. activation protein, β (14-3-3β) | gi|31543974 | Q9CQV8 | ROI | [97] |
| 74 | Vacuolar adenosine triphosphatase subunit B | gi|1184661 | P62814 |  |  |
| 75 | Valosin-containing protein | gi|6005942 | P55072 | ARC, NRF, PBCF | [32,98] |
| 76 | Vimentin | gi|31982755 | P20152 | ARC, NRF, ROI | [32,99] |

List of proteins identified by 2D-DIGE by gel number with cross references to NCBI GI number, Swiss-Prot accession number, and functional protein categories (ARC, actin-related/cytoskeletal; NRF, Nrf2-regulated proteins; PBCF, protease balance/chaperone function; ROI, regulation of inflammation; and RDP, regulatory/ differentiative processes). Classification into Functional Categories was based on information in the references cited.
