# Supplementary material for: Sex differences in the response of the alveolar macrophage proteome to treatment with exogenous surfactant protein-A
Source: Proteome Sci. 2012 Jul 23;10:44. doi: 10.1186/1477-5956-10-44 (PMC3570446; doi:10.1186/1477-5956-10-44)
Supplement: Additional file 4 — Tables A – F. Tables for each protein functional group (Tables A-E) and for all proteins (Table F) summarizing changes of each female group relative to KO baseline values. The data for each functional group (Tables A-E) are extracted from the complete list presented in Table F. [file 1477-5956-10-44-S4.doc]

Reference gel image of mouse alveolar macrophage proteins identified by 2D-DIGE. All identified proteins are circled and numbered. Protein names are listed in below. Some of the proteins may include multiple spots reflecting different isoforms, fragments, or multimers (see also Additional File 3). 1, 65-kDa macrophage protein; 2, Actin related protein 2/3 complex, subunit 5; 3, Actin-related protein 3; 4, Actr2 protein; 5, Alpha-fetoprotein; 6, Annexin A2; 7, Annexin A4; 8, Anxa5 protein; 9, ArsA arsenite transporter, ATP-binding, homolog 1; 10, Atp5b protein; 11, Calpain, small subunit 1; 12, Capping protein (actin filament) muscle Z-line, alpha 2; 13, Capping protein (actin filament) muscle Z-line, beta isoform a; 14, Cathepsin D precursor; 15, Chaperonin subunit 2 (beta); 16, Chia protein; 17, Chitinase 3-like 3 precursor; 18, Chitinase-related protein MCRP; 19, Chloride intracellular channel 1; 20, Chloride intracellular channel 4; 21, CNDP dipeptidase 2; 22, Coactosin-like 1; 23, EF hand domain containing 2; 24, Eno1 protein (Alpha-enolase); 25, Eukaryotic translation initiation factor 5A; 26, Ezrin; 27, F-actin capping protein alpha-1 subunit; 28, Ferritin heavy chain 1; 29, Ferritin light chain 1; 30, Gamma-actin; 31, Gelsolin precursor; 32, Glucose-6-phosphate dehydrogenase X-linked; 33, Guanine deaminase; 34, Heat shock protein 1, beta; 35, Heat shock protein 5 precursor; 36, Heat shock protein 65; 37, Heat shock protein 8; 38, Heat shock protein 90, beta (Grp94), member 1; 39, Hematopoietic cell specific Lyn substrate 1; 40, Heme-binding protein; 41, Heterogeneous nuclear ribonucleoprotein K; 42, High mobility group 1 protein; 43, Hnrpf protein; 44, Kappa-B motif-binding phosphoprotein; 45, Keratin complex 2, basic, gene 8; 46, Keratin type II; 47, Krt13 protein; 48, Laminin receptor; 49, Major vault protein (MVP); 50, Microtubule-associated protein, RP/EB family, member 1; 51, Myosin light chain, regulatory B-like; 52, Nucleophosmin 1; 53, p50b; 54, Peroxiredoxin 2; 55, Prolyl 4-hydroxylase, beta polypeptide precursor; 56, Proteasome (prosome, macropain) 28 subunit, alpha; 57, Proteasome alpha 1 subunit; 58, Protein disulfide isomerase associated 6; 59, Protein disulfide-isomerase A3 precursor; 60, Protein synthesis initiation factor 4A; 61, Purine nucleoside phosphorylase; 62, Put. beta-actin (aa 27-375); 63, Rab GDP dissociation inhibitor beta; 64, Rho GDP dissociation inhibitor (GDI) alpha; 65, Rho, GDP dissociation inhibitor (GDI) beta; 66, Serine (or cysteine) proteinase inhibitor, clade B, member 1a; 67, Stathmin; 68, Superoxide dismutase 1, soluble; 69, Superoxide dismutase 1, soluble; 70, Tropomyosin 3, gamma; 71, Tubulin, beta 5; 72, Tyrosine 3/tryptophan 5 -monooxygenase activation protein, epsilon polypeptide; 73, Tyrosine 3-monooxygenase/tryptophan 5-monooxygenase activation protein, beta polypeptide; 74, Vacuolar adenosine triphosphatase subunit B; 75, Valosin-containing protein; 76, Vimentin.
