# Supplementary material for: Sex differences in the response of the alveolar macrophage proteome to treatment with exogenous surfactant protein-A
Source: Proteome Sci. 2012 Jul 23;10:44. doi: 10.1186/1477-5956-10-44 (PMC3570446; doi:10.1186/1477-5956-10-44)
Supplement: Additional file 5 — Tables A – F. Tables for each protein functional group (Tables A-E) and for all proteins (Table F) summarizing sex differences for each treatment group. The data for each functional group (Tables A-E) are extracted from the complete list presented in Table F. [file 1477-5956-10-44-S5.doc]

**Additional File 5**

**Values for all identified female alveolar macrophage proteins with note of significant changes**.

| **Gel No.** | **Protein Name** | **KO**  **[Group 1]** | **KO 6hr SP-A**  **[Group 2]** | **KO 18 hr SP-A**  **[Group 3]** | **WT**  **[Group 4]** |
| --- | --- | --- | --- | --- | --- |
| 1 | 65-kDa macrophage protein | 6.55 (0.392) | 5.97 (0.400) | 5.97 (0.353) | 6.49 (0.405) |
| 2 | Actin related protein 2/3 complex, subunit 5 | 0.96 (0.055) | 0.92 (0.063) | 0.98 (0.094) | 1.00 (0.032) |
| 3 | Actin-related protein 3 | 1.99 (0.074) | 1.98 (0.021) | 1.98 (0.098) | 2.06 (0.134) |
| 4 | Actr2 protein | 0.90 (0.134) | 0.95 (0.048) | 0.95 (0.086) | 0.98 (0.047) |
| 5 | Alpha-fetoprotein | 7.25 (0.699)^2^ | 10.37 (0.688)^1,4^ | 8.77 (1.312) | 7.32 (0.389)^2^ |
| 6 | Annexin A2 | 2.11 (0.101) | 1.93 (0.135)^4^ | 1.98 (0.107) | 2.11 (0.024)^2^ |
| 7 | Annexin A4 | 0.96 (0.041) | 0.96 (0.074) | 0.93 (0.021) | 0.95 (0.008) |
| 8 | Anxa5 protein | 3.68 (0.233)^4^ | 3.80 (0.074)^3,4^ | 3.50 (0.177)^2,4^ | 4.06 (0.171)^1,2,3^ |
| 9 | ArsA arsenite transporter, ATP-binding, homolog 1 | 1.05 (0.051) | 0.95 (0.094) | 1.03 (0.029) | 1.04 (0.037) |
| 10 | Atp5b protein | 2.03 (0.158) | 2.01 (0.077) | 2.03 (0.038) | 2.01 (0.083) |
| 11 | Calpain, small subunit 1 | 1.06 (0.036)^3^ | 1.07 (0.123) | 0.95 (0.077)^1^ | 1.03 (0.042) |
| 12 | Capping protein (actin filament) muscle Z-line, alpha 2 (CapZ alpha-2) | 1.04 (0.023)^2,3^ | 0.96 (0.055)^1^ | 0.94 (0.051)^1,4^ | 1.03 (0.026)^3^ |
| 13 | Capping protein (actin filament) muscle Z-line, beta isoform a (CapZ beta) | 1.08 (0.044)^2^ | 0.99 (0.043)^1^ | 1.01 (0.048) | 1.05 (0.035) |
| 14 | Cathepsin D precursor | 1.07 (0.076) | 1.08 (0.148) | 0.98 (0.070) | 1.02 (0.018) |
| 15 | Chaperonin subunit 2 (beta) (CCT2) | 2.19 (0.159) | 2.17 (0.263) | 2.31 (0.378) | 2.27 (0.211) |
| 16 | Chia protein | 1.15 (0.246) | 1.10 (0.163) | 1.06 (0.206) | 0.95 (0.076) |
| 17 | Chitinase 3-like 3 precursor (Ym1) | 0.88 (0.039) | 1.56 (0.789) | 1.02 (0.124)^4^ | 0.85 (0.042)^3^ |
| 18 | Chitinase-related protein MCRP | 0.46 (0.096) | 3.74 (4.471) | 0.75 (0.351) | 0.42 (0.026) |
| 19 | Chloride intracellular channel 1 | 2.11 (0.101) | 2.16 (0.202) | 2.07 (0.072) | 2.00 (0.068) |
| 20 | Chloride intracellular channel 4 (mitochondrial) | 1.55 (0.252) | 2.49 (1.115) | 1.65 (0.229) | 1.55 (0.189) |
| 21 | CNDP dipeptidase 2 | 1.99 (0.138) | 1.95 (0.113)^4^ | 1.99 (0.068)^4^ | 2.10 (0.022)^2,3^ |
| 22 | Coactosin-like 1 | 2.25 (0.346) | 2.48 (0.315) | 2.22 (0.214)^4^ | 2.71 (0.217)^3^ |
| 23 | EF hand domain containing 2 | 1.05 (0.028) | 0.99 (0.052) | 1.02 (0.063) | 1.07 (0.041) |
| 24 | Eno1 protein (Alpha-enolase) | 1.85 (0.188)^2^ | 2.15 (0.125)^1^ | 2.13 (0.385) | 1.98 (0.119) |
| 25 | Eukaryotic translation initiation factor 5A | 1.62 (0.188)^3^ | 1.78 (0.284) | 1.95 (0.185)^1^ | 1.87 (0.313) |
| 26 | Ezrin | 5.16 (0.347)^2^ | 4.58 (0.309)^1^ | 4.64 (0.251) | 4.99 (0.238) |
| 27 | F-actin capping protein alpha-1 subunit (CapZ alpha-1) | 1.76 (0.082) | 1.86 (0.030) | 1.82 (0.316) | 1.83 (0.062) |
| 28 | Ferritin heavy chain 1 | 3.16 (0.429) | 3.19 (0.370) | 3.11 (0.389) | 2.85 (0.150) |
| 29 | Ferritin light chain 1 | 2.21 (0.112)^4^ | 2.05 (0.272) | 1.95 (0.303) | 2.05 (0.056)^1^ |
| 30 | Gamma-actin | 17.72 (0.483)^3^ | 17.41 (1.632) | 16.73 (0.333)^1^ | 16.94 (0.457) |
| 31 | Gelsolin precursor | 6.59 (0.423) | 7.29 (0.934) | 6.69 (1.044) | 6.74 (0.353) |
| 32 | Glucose-6-phosphate dehydrogenase X-linked | 1.02 (0.065) | 1.04 (0.058) | 1.05 (0.150) | 1.05 (0.075) |
| 33 | Guanine deaminase | 2.22 (0.215) | 2.26 (0.289) | 2.12 (0.171) | 2.09 (0.079) |
| 34 | Heat shock protein 1, beta (HSP90AB1) | 1.06 (0.019)^2,4^ | 1.01 (0.017)^1^ | 1.04 (0.024)^4^ | 0.99 (0.029)^1,3^ |
| 35 | Heat shock protein 5 precursor (GRP78) | 4.25 (0.203)^4^ | 4.88 (0.753)^4^ | 4.33 (0.497) | 3.88 (0.194)^1,2^ |
| 36 | Heat shock protein 65 (HSP60) | 2.25 (0.231) | 2.20 (0.206) | 2.20 (0.183) | 2.09 (0.153) |
| 37 | Heat shock protein 8 (HSC70; HSC71) | 4.10 (0.179) | 4.23 (0.141)^4^ | 4.04 (0.221) | 3.91 (0.124)^2^ |
| 38 | Heat shock protein 90, beta (Grp94), member 1 | 1.02 (0.042) | 1.04 (0.082) | 0.99 (0.059) | 0.96 (0.026) |
| 39 | Hematopoietic cell specific Lyn substrate 1 | 3.63 (0.280) | 3.41 (0.459) | 3.63 (0.255) | 3.72 (0.337) |
| 40 | Heme-binding protein | 1.21 (0.027)^2,4^ | 1.02 (0.109)^1,4^ | 1.10 (0.127)^4^ | 0.53 (0.057)^1,2,3^ |
| 41 | Heterogeneous nuclear ribonucleoprotein K | 1.18 (0.127)^2^ | 1.00 (0.064)^1,4^ | 1.03 (0.045) | 1.14 (0.085)^2^ |
| 42 | High mobility group 1 protein | 1.01 (0.084) | 0.95 (0.093) | 0.95 (0.089) | 1.02 (0.129) |
| 43 | Hnrpf protein | 2.05 (0.060) | 2.02 (0.051) | 1.93 (0.090) | 1.99 (0.044) |
| 44 | Kappa-B motif-binding phosphoprotein | 3.50 (0.242) | 3.25 (0.198) | 3.20 (0.231) | 3.26 (0.334) |
| 45 | Keratin complex 2, basic, gene 8 | 1.60 (0.215)^4^ | 1.57 (0.149)^4^ | 1.79 (0.240)^4^ | 4.16 (1.027)^1,2,3^ |
| 46 | Keratin type II | 2.13 (0.234) | 2.33 (0.370) | 2.15 (0.396) | 2.12 (0.114) |
| 47 | Krt13 protein | 0.51 (0.088) | 2.04 (1.701) | 0.73 (0.232) | 0.49 (0.041) |
| 48 | Laminin receptor | 1.93 (0.150) | 1.94 (0.103) | 2.07 (0.076)^4^ | 1.91 (0.076)^3^ |
| 49 | Major vault protein (MVP) | 3.38 (0.366) | 3.16 (0.188) | 3.28 (0.143)^4^ | 3.00 (0.127)^3^ |
| 50 | Microtubule-associated protein, RP/EB family, member 1 | 0.95 (0.069) | 0.94 (0.049) | 0.94 (0.041) | 0.98 (0.055) |
| 51 | Myosin light chain, regulatory B-like | 4.62 (0.547) | 4.70 (0.682) | 4.93 (0.954) | 4.37 (0.589) |
| 52 | Nucleophosmin 1 | 4.26 (0.132)^3,4^ | 4.15 (0.153) | 3.98 (0.045)^1,4^ | 4.07 (0.046)^1,3^ |
| 53 | p50b | 1.11 (0.066)^2^ | 0.93 (0.055)^1,4^ | 1.00 (0.094) | 1.05 (0.080)^2^ |
| 54 | Peroxiredoxin 2 | 0.97 (0.109) | 0.97 (0.155) | 1.08 (0.277) | 0.99 (0.116) |
| 55 | Prolyl 4-hydroxylase, beta polypeptide precursor | 3.51 (0.150)^2,3^ | 4.10 (0.255)^1,4^ | 3.91 (0.256)^1,4^ | 3.32 (0.123)^2,3^ |
| 56 | Proteasome (prosome, macropain) 28 subunit, alpha | 1.88 (0.112)^2,4^ | 2.30 (0.319)^1^ | 1.94 (0.054)^4^ | 2.08 (0.077)^1,3^ |
| 57 | Proteasome alpha 1 subunit | 1.03 (0.119) | 1.06 (0.059) | 1.09 (0.123) | 0.94 (0.074) |
| 58 | Protein disulfide isomerase associated 6 (PDI-P5) | 3.67 (0.232) | 3.97 (0.311) | 3.98 (0.402) | 3.76 (0.081) |
| 59 | Protein disulfide-isomerase A3 precursor | 4.11 (0.430) | 4.28 (0.211)^4^ | 4.08 (0.489) | 3.68 (0.087)^2^ |
| 60 | Protein synthesis initiation factor 4A | 1.04 (0.044) | 1.19 (0.260) | 1.11 (0.042)^4^ | 1.01 (0.036)^3^ |
| 61 | Purine nucleoside phosphorylase | 0.80 (0.064)^2,4^ | 1.06 (0.144)^1,3,4^ | 0.82 (0.071)^2,4^ | 1.83 (0.275)^1,2,3^ |
| 62 | Put. beta-actin (aa 27-375) | 2.63 (0.754) | 3.70 (0.580)^3^ | 2.64 (0.565)^2^ | 2.69 (0.639) |
| 63 | Rab GDP dissociation inhibitor beta | 1.05 (0.052) | 1.04 (0.093) | 0.98 (0.022)^4^ | 1.02 (0.021)^3^ |
| 64 | Rho GDP dissociation inhibitor (GDI) alpha | 1.72 (0.023)^3,4^ | 2.47 (0.824) | 1.85 (0.058)^1^ | 1.85 (0.022)^1^ |
| 65 | Rho, GDP dissociation inhibitor (GDI) beta | 2.16 (0.198) | 3.85 (1.807) | 2.66 (0.578) | 2.25 (0.164) |
| 66 | Serine (or cysteine) proteinase inhibitor, clade B, member 1a | 1.84 (0.128) | 1.82 (0.051)^4^ | 1.99 (0.153) | 2.00 (0.120)^2^ |
| 67 | Stathmin | 0.87 (0.077)^3,4^ | 0.97 (0.122) | 1.02 (0.090)^1^ | 1.12 (0.158)^1^ |
| 68 | Superoxide dismutase 1, soluble | 1.89 (0.369) | 2.43 (0.673)^4^ | 1.93 (0.331) | 1.55 (0.172)^2^ |
| 69 | Tropomodulin 3 | 2.97 (0.244) | 2.81 (0.184) | 2.81 (0.166) | 2.70 (0.207) |
| 70 | Tropomyosin 3, gamma | 0.99 (0.074) | 0.87 (0.148) | 0.88 (0.050)^4^ | 1.07 (0.138)^3^ |
| 71 | Tubulin, beta 5 | 2.75 (0.166) | 2.87 (0.153) | 2.82 (0.185) | 2.80 (0.052) |
| 72 | Tyrosine 3/tryptophan 5 -monooxygenase activation protein, ε | 2.01 (0.246) | 1.86 (0.235) | 1.93 (0.196) | 1.98 (0.193) |
| 73 | Tyrosine 3-monooxygenase/tryptophan 5-monooxygenase activation protein, β | 2.57 (0.076) | 3.67 (1.274) | 2.75 (0.291) | 2.48 (0.063) |
| 74 | Vacuolar adenosine triphosphatase subunit B | 3.91 (0.256)^2^ | 4.37 (0.259)^1^ | 4.26 (0.324) | 3.95 (0.238) |
| 75 | Valosin-containing protein | 3.36 (0.362) | 3.16 (0.381) | 3.36 (0.250) | 3.14 (0.271) |
| 76 | Vimentin | 4.13 (0.218)^3^ | 3.87 (0.117) | 3.78 (0.138)^1^ | 3.80 (0.162) |

Values are means ± SD of the normalized volumes for all proteins identified by 2D-DIGE. Significance determined by t-test (p<0.05) for each group (n=4/group). Significantly different from knockout (1), KO 6hr SP-A (2), KO 18hr SP-A (3) and wild-type (4). For proteins with multiple isoforms, the normalized volumes for all isoforms were added together.
