# Supplementary material for: Sex differences in the response of the alveolar macrophage proteome to treatment with exogenous surfactant protein-A
Source: Proteome Sci. 2012 Jul 23;10:44. doi: 10.1186/1477-5956-10-44 (PMC3570446; doi:10.1186/1477-5956-10-44)
Supplement: Additional file 6 — Title: MIAPE: Gel Electrophoresis. Description: File containing Minimum Information About a Proteomics Experiment – Gel Electrophoresis in the format recommended by the Human Proteome Organization Proteomic Standards Initiative. [file 1477-5956-10-44-S6.doc]

**Additional File 6**

**Table A. Changes in protease balance/chaperone function proteins for female mice (as compared to KO baseline from same sex).**

| **Gel No.** | **Protein Name** |  | **KO 6 hr SP-A**  **Female** |  | | | **KO 18 hr SP-A**  **Female** |  | | **WT**  **Female** |
| --- | --- | --- | --- | --- | --- | --- | --- | --- | --- | --- |
| 11 | Calpain, small subunit 1 |  | **↑** |  | | | **↓*** |  | | **↓** |
| 14 | Cathepsin D precursor |  | **↑** |  | | | **↓** |  | | **↓** |
| 15 | Chaperonin subunit 2 (beta) |  | **↓** |  | | | **↑** |  | | **↑** |
| 19 | Chloride intracellular channel 1 |  | **↑** | |  | **↓** | |  | **↓** | |
| 21 | CNDP dipeptidase 2 |  | **↓** |  | | | **↑** |  | | **↑** |
| 22 | Coactosin-like 1 |  | **↑** | |  | **↓** | |  | **↑** | |
| 24 | Eno1 protein (Alpha-enolase) |  | **↑*** |  | | | **↑** |  | | **↑** |
| 34 | Heat shock protein 1, beta (HSP90AB1) |  | **↓*** |  | | | **↓** |  | | **↓*** |
| 35 | Heat shock protein 5 precursor (GRP78) |  | **↑** |  | | | **↑** |  | | **↓*** |
| 36 | Heat shock protein 65 (HSP60) |  | **↓** |  | | | **↓** |  | | **↓** |
| 37 | Heat shock protein 8 (HSC70; HSC71) |  | **↑** |  | | | **↓** |  | | **↓** |
| 38 | Heat shock protein 90, beta (Grp94), member 1 |  | **↑** |  | | | **↓** |  | | **↓** |
| 55 | Prolyl 4-hydroxylase, beta polypeptide precursor |  | **↑*** | |  | **↑*** | |  | **↓** | |
| 56 | Proteasome (prosome, macropain) 28 subunit, alpha |  | **↑*** |  | | | **↑** |  | | **↑*** |
| 57 | Proteasome alpha 1 subunit |  | **↑** |  | | | **↑** |  | | **↓** |
| 58 | Protein disulfide isomerase associated 6 |  | **↑** |  | | | **↑** |  | | **↑** |
| 59 | Protein disulfide-isomerase A3 precursor |  | **↑** |  | | | **↓** |  | | **↓** |
| 66 | Serine (or cysteine) proteinase inhibitor, clade B, member 1a |  | **↓** |  | | | **↑** |  | | **↑** |
| 75 | Valosin-containing protein |  | **↓** |  | | | **↓** |  | | **↓** |
|  | **Total changes (including significant)** |  | **6↓, 13↑** | |  | **10↓, 9↑** | |  | **12↓, 7↑** | |
|  | **Total significant changes** |  | **1↓*, 3↑*** | |  | **1↓*, 1↑*** | |  | **2↓*, 1↑*** | |

Comparison of mean normalized volumes for proteins from female mice for KO to KO 6 hr SP-A, KO 18 hr SP-A, and wild-type mice of the same sex. Increased compared to KO (**↑**), decreased compared to KO (**↓**), determined to be significant (p<0.05) by t-test (*****).

**Additional File 6**

**Table B. Changes in actin-related proteins for female mice (as compared to KO baseline from same sex).**

| **Gel No.** | **Protein Name** |  | **KO 6 hr SP-A**  **Female** |  | | | **KO 18 hr SP-A**  **Female** |  | | **WT**  **Female** |
| --- | --- | --- | --- | --- | --- | --- | --- | --- | --- | --- |
| 1 | 65-kDa macrophage protein |  | **↓** |  | | | **↓** |  | | **↓** |
| 2 | Actin related protein 2/3 complex, subunit 5 |  | **↓** |  | | | **↑** |  | | **↑** |
| 3 | Actin-related protein 3 |  | **↓** |  | | | **↓** |  | | **↑** |
| 4 | Actr2 protein |  | **↑** |  | | | **↑** |  | | **↑** |
| 6 | Annexin A2 |  | **↓** |  | | | **↓** |  | | **↓** |
| 11 | Calpain, small subunit 1 |  | **↑** | |  | **↓*** | |  | **↓** | |
| 12 | Capping protein (actin filament) muscle Z-line, alpha 2 (capZ alpha-2) |  | **↓*** |  | | | **↓*** |  | | **↓** |
| 13 | Capping protein (actin filament) muscle Z-line, beta isoform a (capZ beta) |  | **↓*** |  | | | **↓** |  | | **↓** |
| 15 | Chaperonin subunit 2 (beta) (CCT2) |  | **↓** | |  | **↑** | |  | **↑** | |
| 19 | Chloride intracellular channel 1 |  | **↑** |  | | | **↓** |  | | **↓** |
| 20 | Chloride intracellular channel 4 (mitochondrial) |  | **↑** |  | | | **↑** |  | | **↑** |
| 22 | Coactosin-like 1 |  | **↑** |  | | | **↓** |  | | **↑** |
| 24 | Eno1 protein (Alpha-enolase) |  | **↑*** | |  | **↑** | |  | **↑** | |
| 25 | Eukaryotic translation initiation factor 5A |  | **↑** |  | | | **↑*** |  | | **↑** |
| 26 | Ezrin |  | **↓*** |  | | | **↓** |  | | **↓** |
| 27 | F-actin capping protein alpha-1 subunit (capZ alpha-1) |  | **↑** |  | | | **↑** |  | | **↑** |
| 30 | Gamma-actin |  | **↓** |  | | | **↓*** |  | | **↓** |
| 31 | Gelsolin precursor |  | **↑** |  | | | **↑** |  | | **↑** |
| 33 | Guanine deaminase |  | **↑** | |  | **↓** | |  | **↓** | |
| 34 | Heat shock protein 1, beta |  | **↓*** |  | | | **↓** |  | | **↓*** |
| 39 | Hematopoietic cell specific Lyn substrate 1 |  | **↓** |  | | | **↓** |  | | **↑** |
| 45 | Keratin complex 2, basic, gene 8 |  | **↓** |  | | | **↑** |  | | **↑*** |
| 46 | Keratin type II |  | **↑** |  | | | **↑** |  | | **↓** |
| 47 | Krt13 protein |  | **↑** | |  | **↑** | |  | **↓** | |
| 49 | Major vault protein (MVP) |  | **↓** | |  | **↓** | |  | **↓** | |
| 50 | Microtubule-associated protein, RP/EB family, member 1 |  | **↓** |  | | | **↓** |  | | **↑** |
| 51 | Myosin light chain, regulatory B-like |  | **↑** |  | | | **↑** |  | | **↓** |
| 53 | p50b; Leukocyte-specific protein 1 (LSP1) |  | **↓*** | |  | **↓** | |  | **↓** | |
| 62 | Put. beta-actin (aa 27-375) |  | **↑** |  | | | **↑** |  | | **↑** |
| 63 | Rab GDP dissociation inhibitor beta |  | **↓** |  | | | **↓** |  | | **↓** |
| 64 | Rho GDP dissociation inhibitor (GDI) alpha |  | **↑** |  | | | **↑*** |  | | **↑*** |
| 65 | Rho, GDP dissociation inhibitor (GDI) beta |  | **↑** |  | | | **↑** |  | | **↑** |
| 67 | Stathmin |  | **↑** |  | | | **↑*** |  | | **↑*** |
| 69 | Tropomodulin 3 |  | **↓** |  | | | **↓** |  | | **↓** |
| 70 | Tropomyosin 3, gamma |  | **↓** |  | | | **↓** |  | | **↑** |
| 71 | Tubulin, beta 5 |  | **↑** |  | | | **↑** |  | | **↑** |
| 75 | Valosin-containing protein |  | **↓** |  | | | **↓** |  | | **↓** |
| 76 | Vimentin |  | **↓** |  | | | **↓*** |  | | **↓** |
|  | **Total changes (including significant)** |  | **20↓, 18↑** | |  | **21↓, 17↑** | |  | **19↓, 19↑** | |
|  | **Total significant changes** |  | **5↓*, 1↑*** | |  | **4↓*, 3↑*** | |  | **1↓*, 3↑*** | |

Comparison of mean normalized volumes for proteins from female mice for KO to KO 6 hr SP-A, KO 18 hr SP-A, and wild-type mice of the same sex. Increased compared to KO (**↑**), decreased compared to KO (**↓**), determined to be significant (p<0.05) by t-test (*****).

**Additional File 6**

**Table C. Changes in Nrf-2 regulated (NRF) proteins for female mice (as compared to KO baseline from the same sex**).

| **Gel No.** | **Protein Name** |  | **KO 6 hr SP-A**  **Female** |  | | | **KO 18 hr SP-A**  **Female** |  | | **WT**  **Female** |
| --- | --- | --- | --- | --- | --- | --- | --- | --- | --- | --- |
| 1 | 65-kDa macrophage protein |  | **↓** |  | | | **↓** |  | | **↓** |
| 14 | Cathepsin D precursor |  | **↑** | |  | **↓** | |  | **↓** | |
| 20 | Chloride intracellular channel 4 (mitochondrial) |  | **↑** |  | | | **↑** |  | | **↑** |
| 28 | Ferritin heavy chain 1 |  | **↑** | |  | **↓** | |  | **↓** | |
| 29 | Ferritin light chain 1 |  | **↓** | |  | **↓** | |  | **↓*** | |
| 30 | Gamma-actin |  | **↓** |  | | | **↓*** |  | | **↓** |
| 31 | Gelsolin precursor |  | **↑** |  | | | **↑** |  | | **↑** |
| 32 | Glucose-6-phosphate dehydrogenase X-linked |  | **↑** | |  | **↑** | |  | **↑** | |
| 34 | Heat shock protein 1, beta (HSP90AB1) |  | **↓*** |  | | | **↓** |  | | **↓*** |
| 35 | Heat shock protein 5 precursor (GRP78) |  | **↑** | |  | **↑** | |  | **↓*** | |
| 38 | Heat shock protein 90, beta (Grp94), member 1 |  | **↑** | |  | **↓** | |  | **↓** | |
| 45 | Keratin complex 2, basic, gene 8 |  | **↓** |  | | | **↑** |  | | **↑*** |
| 47 | Krt13 protein |  | **↑** | |  | **↑** | |  | **↓** | |
| 54 | Peroxiredoxin 2 |  | **↓** | |  | **↑** | |  | **↑** | |
| 57 | Proteasome alpha 1 subunit |  | **↑** | |  | **↑** | |  | **↓** | |
| 60 | Protein synthesis initiation factor 4A |  | **↑** | |  | **↑** | |  | **↓** | |
| 62 | Put. beta-actin (aa 27-375) |  | **↑** |  | | | **↑** |  | | **↑** |
| 68 | Superoxide dismutase 1, soluble |  | **↑** | |  | **↑** | |  | **↓** | |
| 71 | Tubulin, beta 5 |  | **↑** |  | | | **↑** |  | | **↑** |
| 75 | Valosin-containing protein |  | **↓** |  | | | **↓** |  | | **↓** |
| 76 | Vimentin |  | **↓** |  | | | **↓*** |  | | **↓** |
|  | **Total changes (including significant)** |  | **8↓, 13↑** | |  | **9↓, 12↑** | |  | **14↓, 7↑** | |
|  | **Total significant changes** |  | **1↓*** | |  | **2↓*** | |  | **3↓*, 1↑*** | |

Comparison of mean normalized volumes for proteins from female mice for KO to KO 6 hr SP-A, KO 18 hr SP-A, and wild-type mice of the same sex. Increased compared to KO (**↑**), decreased compared to KO (**↓**), determined to be significant (p<0.05) by t-test (*****).

**Additional File 6**

**Table D. Changes in regulatory/differentiative processes proteins for f**emale mice (as compared to KO baseline from same sex).

| **Gel No.** | **Protein Name** |  | **KO 6 hr SP-A**  **Female** |  | | | **KO 18 hr SP-A**  **Female** |  | | **WT**  **Female** |
| --- | --- | --- | --- | --- | --- | --- | --- | --- | --- | --- |
| 25 | Eukaryotic translation initiation factor 5A |  | **↑** |  | | | **↑*** |  | | **↑** |
| 41 | Heterogeneous nuclear ribonucleoprotein K |  | **↓*** |  | | | **↓** |  | | **↓** |
| 42 | High mobility group 1 protein |  | **↓** |  | | | **↓** |  | | **↑** |
| 43 | Hnrpf protein |  | **↓** |  | | | **↓** |  | | **↓** |
| 44 | Kappa-B motif-binding phosphoprotein |  | **↓** |  | | | **↓** |  | | **↓** |
| 52 | Nucleophosmin 1 |  | **↓** |  | | | **↓*** |  | | **↓*** |
| 53 | p50b; Leukocyte-specific protein 1 (LSP1) |  | **↓*** |  | | | **↓** |  | | **↓** |
| 60 | Protein synthesis initiation factor 4A |  | **↑** |  | | | **↑** |  | | **↓** |
|  | **Total changes (including significant)** |  | **6↓, 2↑** | |  | **6↓, 2↑** | |  | **6↓, 2↑** | |
|  | **Total significant changes** |  | **2↓*** | |  | **1↓*, 1↑*** | |  | **1↓*** | |

Comparison of mean normalized volumes for proteins from female mice for KO to KO 6 hr SP-A, KO 18 hr SP-A, and wild-type mice of the same sex. Increased compared to KO (**↑**), decreased compared to KO (**↓**), determined to be significant (p<0.05) by t-test (*****).

**Additional File 6**

**Table E. Changes in regulation of inflammation proteins for f**emale mice (as compared to KO baseline from same sex).

| **Gel No.** | **Protein Name** |  | **KO 6 hr SP-A**  **Female** |  | | | **KO 18 hr SP-A**  **Female** |  | | **WT**  **Female** |
| --- | --- | --- | --- | --- | --- | --- | --- | --- | --- | --- |
| 5 | Alpha-fetoprotein |  | **↑*** |  | | | **↑** |  | | **↑** |
| 6 | Annexin A2 |  | **↓** |  | | | **↓** |  | | **↓** |
| 7 | Annexin A4 |  | **↑** | |  | **↓** | |  | **↓** | |
| 16 | Chia protein |  | **↓** |  | | | **↓** |  | | **↓** |
| 17 | Chitinase 3-like 3 precursor (Ym1) |  | **↑** |  | | | **↑** |  | | **↓** |
| 18 | Chitinase-related protein MCRP |  | **↑** |  | | | **↑** |  | | **↓** |
| 24 | Eno1 protein (Alpha-enolase) |  | **↑*** |  | | | **↑** |  | | **↑** |
| 25 | Eukaryotic translation initiation factor 5A |  | **↑** |  | | | **↑*** |  | | **↑** |
| 34 | Heat shock protein 1, beta (HSP90AB1) |  | **↓*** |  | | | **↓** |  | | **↓*** |
| 35 | Heat shock protein 5 precursor (GRP78) |  | **↑** | |  | **↑** | |  | **↓*** | |
| 36 | Heat shock protein 65 (HSP60) |  | **↓** | |  | **↓** | |  | **↓** | |
| 37 | Heat shock protein 8 (HSC70; HSC71) |  | **↑** | |  | **↓** | |  | **↓** | |
| 38 | Heat shock protein 90, beta (Grp94), member 1 |  | **↑** | |  | **↓** | |  | **↓** | |
| 39 | Hematopoietic cell specific Lyn substrate 1 |  | **↓** |  | | | **↓** |  | | **↑** |
| 40 | Heme-binding protein |  | **↓*** |  | | | **↓** |  | | **↓*** |
| 42 | High mobility group 1 protein |  | **↓** |  | | | **↓** |  | | **↑** |
| 53 | p50b; Leukocyte-specific protein 1 (LSP1) |  | **↓*** | |  | **↓** | |  | **↓** | |
| 72 | Tyrosine 3/tryptophan 5 -monooxygenase activation protein, ε |  | **↓** |  | | | **↓** |  | | **↓** |
| 73 | Tyrosine 3-monooxygenase/tryptophan 5-monooxygenase activation protein, β |  | **↑** |  | | | **↑** |  | | **↓** |
| 76 | Vimentin |  | **↓** | |  | **↓*** | |  | **↓** | |
|  | **Total changes (including significant)** |  | **10↓, 10↑** | |  | **13↓, 7↑** | |  | **15↓, 5↑** | |
|  | **Total significant changes** |  | **3↓*, 2↑*** | |  | **1↓*, 1↑*** | |  | **3↓*** | |

Comparison of mean normalized volumes for proteins from female mice for KO to KO 6 hr SP-A, KO 18 hr SP-A, and wild-type mice of the same sex. Increased compared to KO (**↑**), decreased compared to KO (**↓**), determined to be significant (p<0.05) by t-test (*****).

**Additional File 6**

**Table F. Changes in all proteins for f**emale mice (as compared to KO baseline from same sex).

| **Gel No.** | **Protein Name** |  | **KO 6 hr SP-A**  **Female** |  | **KO 18 hr SP-A**  **Female** |  | **WT**  **Female** |
| --- | --- | --- | --- | --- | --- | --- | --- |
| 1 | 65-kDa macrophage protein |  | **↓** |  | **↓** |  | **↓** |
| 2 | Actin related protein 2/3 complex, subunit 5 |  | **↓** |  | **↑** |  | **↑** |
| 3 | Actin-related protein 3 |  | **↓** |  | **↓** |  | **↑** |
| 4 | Actr2 protein |  | **↑** |  | **↑** |  | **↑** |
| 5 | Alpha-fetoprotein |  | **↑*** |  | **↑** |  | **↑** |
| 6 | Annexin A2 |  | **↓** |  | **↓** |  | **↓** |
| 7 | Annexin A4 |  | **↑** |  | **↓** |  | **↓** |
| 8 | Anxa5 protein |  | **↑** |  | **↓** |  | **↑*** |
| 9 | ArsA arsenite transporter, ATP-binding, homolog 1 |  | **↓** |  | **↓** |  | **↓** |
| 10 | Atp5b protein |  | **↓** |  | **↓** |  | **↓** |
| 11 | Calpain, small subunit 1 |  | **↑** |  | **↓*** |  | **↓** |
| 12 | Capping protein (actin filament) muscle Z-line, alpha 2 (CapZ alpha-2) |  | **↓*** |  | **↓*** |  | **↓** |
| 13 | Capping protein (actin filament) muscle Z-line, beta isoform a (CapZ beta) |  | **↓*** |  | **↓** |  | **↓** |
| 14 | Cathepsin D precursor |  | **↑** |  | **↓** |  | **↓** |
| 15 | Chaperonin subunit 2 (beta) (CCT2) |  | **↓** |  | **↑** |  | **↑** |
| 16 | Chia protein |  | **↓** |  | **↓** |  | **↓** |
| 17 | Chitinase 3-like 3 precursor (Ym1) |  | **↑** |  | **↑** |  | **↓** |
| 18 | Chitinase-related protein MCRP |  | **↑** |  | **↑** |  | **↓** |
| 19 | Chloride intracellular channel 1 |  | **↑** |  | **↓** |  | **↓** |
| 20 | Chloride intracellular channel 4 (mitochondrial) |  | **↑** |  | **↑** |  | **↑** |
| 21 | CNDP dipeptidase 2 |  | **↓** |  | **↑** |  | **↑** |
| 22 | Coactosin-like 1 |  | **↑** |  | **↓** |  | **↑** |
| 23 | EF hand domain containing 2 |  | **↓** |  | **↓** |  | **↑** |
| 24 | Eno1 protein (Alpha-enolase) |  | **↑*** |  | **↑** |  | **↑** |
| 25 | Eukaryotic translation initiation factor 5A |  | **↑** |  | **↑*** |  | **↑** |
| 26 | Ezrin |  | **↓*** |  | **↓** |  | **↓** |
| 27 | F-actin capping protein alpha-1 subunit (CapZ alpha-1) |  | **↑** |  | **↑** |  | **↑** |
| 28 | Ferritin heavy chain 1 |  | **↑** |  | **↓** |  | **↓** |
| 29 | Ferritin light chain 1 |  | **↓** |  | **↓** |  | **↓*** |
| 30 | Gamma-actin |  | **↓** |  | **↓*** |  | **↓** |
| 31 | Gelsolin precursor |  | **↑** |  | **↑** |  | **↑** |
| 32 | Glucose-6-phosphate dehydrogenase X-linked |  | **↑** |  | **↑** |  | **↑** |
| 33 | Guanine deaminase |  | **↑** |  | **↓** |  | **↓** |
| 34 | Heat shock protein 1, beta (HSP90AB1) |  | **↓*** |  | **↓** |  | **↓*** |
| 35 | Heat shock protein 5 precursor (GRP78) |  | **↑** |  | **↑** |  | **↓*** |
| 36 | Heat shock protein 65 (HSP60) |  | **↓** |  | **↓** |  | **↓** |
| 37 | Heat shock protein 8 (HSC70; HSC71) |  | **↑** |  | **↓** |  | **↓** |
| 38 | Heat shock protein 90, beta (Grp94), member 1 |  | **↑** |  | **↓** |  | **↓** |
| 39 | Hematopoietic cell specific Lyn substrate 1 |  | **↓** |  | **↓** |  | **↑** |
| 40 | Heme-binding protein |  | **↓*** |  | **↓** |  | **↓*** |
| 41 | Heterogeneous nuclear ribonucleoprotein K |  | **↓*** |  | **↓** |  | **↓** |
| 42 | High mobility group 1 protein |  | **↓** |  | **↓** |  | **↑** |
| 43 | Hnrpf protein |  | **↓** |  | **↓** |  | **↓** |
| 44 | Kappa-B motif-binding phosphoprotein |  | **↓** |  | **↓** |  | **↓** |
| 45 | Keratin complex 2, basic, gene 8 |  | **↓** |  | **↑** |  | **↑*** |
| 46 | Keratin type II |  | **↑** |  | **↑** |  | **↓** |
| 47 | Krt13 protein |  | **↑** |  | **↑** |  | **↓** |
| 48 | Laminin receptor |  | **↑** |  | **↑** |  | **↓** |
| 49 | Major vault protein (MVP) |  | **↓** |  | **↓** |  | **↓** |
| 50 | Microtubule-associated protein, RP/EB family, member 1 |  | **↓** |  | **↓** |  | **↑** |
| 51 | Myosin light chain, regulatory B-like |  | **↑** |  | **↑** |  | **↓** |
| 52 | Nucleophosmin 1 |  | **↓** |  | **↓*** |  | **↓*** |
| 53 | p50b |  | **↓*** |  | **↓** |  | **↓** |
| 54 | Peroxiredoxin 2 |  | **↓** |  | **↑** |  | **↑** |
| 55 | Prolyl 4-hydroxylase, beta polypeptide precursor |  | **↑*** |  | **↑*** |  | **↓** |
| 56 | Proteasome (prosome, macropain) 28 subunit, alpha |  | **↑*** |  | **↑** |  | **↑*** |
| 57 | Proteasome alpha 1 subunit |  | **↑** |  | **↑** |  | **↓** |
| 58 | Protein disulfide isomerase associated 6 (PDI-P5) |  | **↑** |  | **↑** |  | **↑** |
| 59 | Protein disulfide-isomerase A3 precursor |  | **↑** |  | **↓** |  | **↓** |
| 60 | Protein synthesis initiation factor 4A |  | **↑** |  | **↑** |  | **↓** |
| 61 | Purine nucleoside phosphorylase |  | **↑*** |  | **↑** |  | **↑*** |
| 62 | Put. beta-actin (aa 27-375) |  | **↑** |  | **↑** |  | **↑** |
| 63 | Rab GDP dissociation inhibitor beta |  | **↓** |  | **↓** |  | **↓** |
| 64 | Rho GDP dissociation inhibitor (GDI) alpha |  | **↑** |  | **↑*** |  | **↑*** |
| 65 | Rho, GDP dissociation inhibitor (GDI) beta |  | **↑** |  | **↑** |  | **↑** |
| 66 | Serine (or cysteine) proteinase inhibitor, clade B, member 1a |  | **↓** |  | **↑** |  | **↑** |
| 67 | Stathmin |  | **↑** |  | **↑*** |  | **↑*** |
| 68 | Superoxide dismutase 1, soluble |  | **↑** |  | **↑** |  | **↓** |
| 69 | Tropomodulin 3 |  | **↓** |  | **↓** |  | **↓** |
| 70 | Tropomyosin 3, gamma |  | **↓** |  | **↓** |  | **↑** |
| 71 | Tubulin, beta 5 |  | **↑** |  | **↑** |  | **↑** |
| 72 | Tyrosine 3/tryptophan 5 -monooxygenase activation protein, ε |  | **↓** |  | **↓** |  | **↓** |
| 73 | Tyrosine 3-monooxygenase/tryptophan 5-monooxygenase activation protein, β |  | **↑** |  | **↑** |  | **↓** |
| 74 | Vacuolar adenosine triphosphatase subunit B |  | **↑*** |  | **↑** |  | **↑** |
| 75 | Valosin-containing protein |  | **↓** |  | **↓** |  | **↓** |
| 76 | Vimentin |  | **↓** |  | **↓*** |  | **↓** |
|  | **Total changes** |  | **36↓, 40↑** |  | **41↓, 35↑** |  | **45↓, 31↑** |
|  | **Total significant changes** |  | **7↓*, 6↑*** |  | **5↓*, 4↑*** |  | **5↓*, 6↑*** |

Comparison of mean normalized volumes (see Additional File 5) for proteins from female KO mice to KO 6 hr SP-A, KO 18 hr SP-A, and WT mice. Increased compared to KO(↑), decreased compared to KO(↓), determined to be significant (p<0.05) by t-test (*).
