# Supplementary material for: Sex differences in the response of the alveolar macrophage proteome to treatment with exogenous surfactant protein-A
Source: Proteome Sci. 2012 Jul 23;10:44. doi: 10.1186/1477-5956-10-44 (PMC3570446; doi:10.1186/1477-5956-10-44)
Supplement: Additional file 7 — Title: MIAPE: Gel Informatics. Description: File containing Minimum Information About a Proteomics Experiment – Gel Informatics in the format recommended by the Human Proteome Organization Proteomic Standards Initiative. [file 1477-5956-10-44-S7.doc]

**Additional File 7**

**Table A. Changes in protease balance/chaperone function proteins from Male to Female mice in the same group.**

| **Gel No.** | **Protein Name** | **KO** | **KO 6 hr SP-A** | **KO 18 hr SP-A** | **WT** |
| --- | --- | --- | --- | --- | --- |
| 11 | Calpain, small subunit 1 | **↓** | **↑** | **↓** | **↓** |
| 14 | Cathepsin D precursor | **↓** | **↑** | **↓** | **↓** |
| 15 | Chaperonin subunit 2 (beta) (CCT2) | **↑** | **↑** | **↑** | **↑*** |
| 19 | Chloride intracellular channel 1 | **↓** | **↑** | **↑** | **UN** |
| 21 | CNDP dipeptidase 2 | **↓** | **↑** | **↑** | **↑** |
| 22 | Coactosin-like 1 | **↓** | **↓** | **↓*** | **↓** |
| 24 | Eno1 protein (Alpha-enolase) | **↓** | **↑** | **↑** | **↑** |
| 34 | Heat shock protein 1, beta (HSP90AB1) | **↑*** | **↑** | **↑*** | **↑** |
| 35 | Heat shock protein 5 precursor (GRP78) | **↑*** | **↑** | **↑*** | **↑** |
| 36 | Heat shock protein 65 (HSP60) | **↑** | **↑** | **↑*** | **↑*** |
| 37 | Heat shock protein 8 (HSC70; HSC71) | **↑** | **↑** | **↓** | **↓** |
| 38 | Heat shock protein 90, beta (Grp94), member 1 | **↓** | **↑** | **↑*** | **↑** |
| 52 | Nucleophosmin 1 | **↓** | **↑** | **↑** | **↑** |
| 56 | Proteasome (prosome, macropain) 28 subunit, alpha | **↓** | **↑** | **↓** | **↓** |
| 57 | Proteasome alpha 1 subunit | **↓** | **↑** | **↑** | **↓** |
| 58 | Protein disulfide isomerase associated 6 (PDI-P5) | **↓** | **↑** | **↑** | **↑** |
| 59 | Protein disulfide-isomerase A3 precursor | **↑** | **↑*** | **↑** | **↑** |
| 66 | Serine (or cysteine) proteinase inhibitor, clade B, member 1a | **↓** | **↓** | **↓** | **UN** |
| 75 | Valosin-containing protein | **↑** | **↑** | **↑*** | **↑*** |
|  | **Total (including significant) changes** | **12↓, 7↑** | **2↓, 17↑** | **6↓, 13↑** | **6↓,11↑,2UN** |
|  | **Total significant changes** | **2↑*** | **1↑*** | **1↓*, 5↑*** | **3↑*** |

Comparison of mean normalized volumes for proteins from Male to Female mice in the same group (KO, KO 6 hr SP-A, KO 18 hr SP-A, and WT). Higher in female (**↑**), lower in female (**↓**), unchanged (UN), determined to be significant (p<0.05) by t-test (*****).

**Additional File 7**

**Table B. Changes in actin-related/cytoskeletal proteins from Male to Female mice in the same group.**

| **Gel No.** | **Protein Name** | **KO** | **KO 6 hr SP-A** | **KO 18 hr SP-A** | **WT** |
| --- | --- | --- | --- | --- | --- |
| 1 | 65-kDa macrophage protein | **↑** | **↑** | **↑** | **↑** |
| 2 | Actin related protein 2/3 complex, subunit 5 | **UN** | **↓** | **↑** | **UN** |
| 3 | Actin-related protein 3 | **↓** | **↑** | **↑** | **↑** |
| 4 | Actr2 protein | **↓** | **↑** | **↓** | **↑** |
| 6 | Annexin A2 | **↓*** | **↓** | **↓** | **UN** |
| 11 | Calpain, small subunit 1 | **↓** | **↑** | **↓** | **↓** |
| 12 | Capping protein (actin filament) muscle Z-line, alpha 2 (CapZ alpha-2) | **↓** | **↓** | **↓*** | **↓** |
| 13 | Capping protein (actin filament) muscle Z-line, beta isoform a (CapZ beta) | **↓** | **↓** | **↓** | **↓** |
| 15 | Chaperonin subunit 2 (beta) (CCT2) | **↑** | **↑** | **↑** | **↑*** |
| 19 | Chloride intracellular channel 1 | **↓** | **↑** | **↑** | **UN** |
| 20 | Chloride intracellular channel 4 (mitochondrial) | **↓** | **↑** | **↓*** | **↓** |
| 22 | Coactosin-like 1 | **↓** | **↓** | **↓*** | **↓** |
| 24 | Eno1 protein (Alpha-enolase) | **↓** | **↑** | **↑** | **↑** |
| 25 | Eukaryotic translation initiation factor 5A | **↓** | **↓** | **↓** | **↑** |
| 26 | Ezrin | **↓** | **↓** | **↓** | **↑** |
| 27 | F-actin capping protein alpha-1 subunit (CapZ alpha-1) | **↓** | **↓** | **↓** | **↓** |
| 30 | Gamma-actin | **↓** | **↓** | **↑** | **↓** |
| 31 | Gelsolin precursor | **↑** | **↑** | **↑** | **↑** |
| 33 | Guanine deaminase | **↑*** | **↑*** | **↑*** | **↑*** |
| 34 | Heat shock protein 1, beta (HSP90AB1) | **↑*** | **↑** | **↑*** | **↑** |
| 39 | Hematopoietic cell specific Lyn substrate 1 | **↓** | **↓** | **↓** | **↓** |
| 45 | Keratin complex 2, basic, gene 8 | **↓** | **↓** | **↑** | **↓** |
| 46 | Keratin type II | **↓** | **↑** | **↑** | **↓** |
| 47 | Krt13 protein | **↓** | **↑** | **UN** | **↓** |
| 49 | Major vault protein (MVP) | **↑** | **↑** | **↑*** | **↑*** |
| 50 | Microtubule-associated protein, RP/EB family, member 1 | **↓** | **↓** | **↑*** | **↑*** |
| 51 | Myosin light chain, regulatory B-like | **↓** | **↓** | **↑** | **↓** |
| 53 | p50b, Leukocyte specific protein 1 (LSP1) | **↓** | **↓** | **↓** | **↓** |
| 62 | Put. beta-actin (aa 27-375) | **↓** | **↓** | **↓*** | **↓** |
| 63 | Rab GDP dissociation inhibitor beta | **↓** | **↑** | **↑** | **↑*** |
| 64 | Rho GDP dissociation inhibitor (GDI) alpha | **↓** | **↑** | **↑*** | **↑*** |
| 65 | Rho, GDP dissociation inhibitor (GDI) beta | **↑** | **↑** | **↑** | **↑** |
| 67 | Stathmin | **UN** | **↑** | **↑** | **↓** |
| 69 | Tropomodulin 3 | **↓** | **↓*** | **↓*** | **↓** |
| 70 | Tropomyosin 3, gamma | **↓** | **↓** | **↓*** | **↓** |
| 71 | Tubulin, beta 5 | **↓** | **↓*** | **↓** | **↓*** |
| 75 | Valosin-containing protein | **↑** | **↑** | **↑*** | **↑*** |
| 76 | Vimentin | **↓** | **↓*** | **↓*** | **↓** |
|  | **Total (including significant) changes** | **28↓,8↑,2UN** | **19↓, 19↑** | **17↓,20↑,1UN** | **19↓,16↑,3UN** |
|  | **Total significant changes** | **1↓*, 2↑*** | **3↓*, 1↑*** | **7↓*, 6↑*** | **1↓*, 7↑*** |

Comparison of mean normalized volumes for proteins from Male to Female mice in the same group (KO, KO 6 hr SP-A, KO 18 hr SP-A, and WT). Higher in female (**↑**), lower in female (**↓**), unchanged (UN), determined to be significant (p<0.05) by t-test (*****).

**Additional File 7**

**Table C. Changes in Nrf-2 related alveolar macrophage proteins from Male to Female mice in the same group.**

| **Gel No.** | **Protein Name** | **KO** | **KO 6 hr SP-A** | **KO 18 hr SP-A** | **WT** |
| --- | --- | --- | --- | --- | --- |
| 1 | 65-kDa macrophage protein | **↑** | **↑** | **↑** | **↑** |
| 14 | Cathepsin D precursor | **↓** | **↑** | **↓** | **↓** |
| 20 | Chloride intracellular channel 4 (mitochondrial) | **↓** | **↑** | **↓*** | **↓** |
| 28 | Ferritin heavy chain 1 | **↑** | **↑** | **↓** | **↓** |
| 29 | Ferritin light chain 1 | **↓** | **↑** | **↓** | **↓** |
| 30 | Gamma-actin | **↓** | **↓** | **↑** | **↓** |
| 31 | Gelsolin precursor | **↑** | **↑** | **↑** | **↑** |
| 32 | Glucose-6-phosphate dehydrogenase X-linked | **↓** | **↑** | **↑** | **↑** |
| 34 | Heat shock protein 1, beta (HSP90AB1) | **↑*** | **↑** | **↑*** | **↑** |
| 35 | Heat shock protein 5 precursor (GRP78) | **↑*** | **↑** | **↑*** | **↑** |
| 38 | Heat shock protein 90, beta (Grp94), member 1 | **↓** | **↑** | **↑*** | **↑** |
| 45 | Keratin complex 2, basic, gene 8 | **↓** | **↓** | **↑** | **↓** |
| 47 | Krt13 protein | **↓** | **↑** | **UN** | **↓** |
| 54 | Peroxiredoxin 2 | **↓** | **↓** | **↑** | **↓** |
| 57 | Proteasome alpha 1 subunit | **↓** | **↑** | **↑** | **↓** |
| 60 | Protein synthesis initiation factor 4A | **↓** | **↑** | **↑*** | **↑*** |
| 62 | Put. beta-actin (aa 27-375) | **↓** | **↓** | **↓*** | **↓** |
| 68 | Superoxide dismutase 1, soluble | **↓** | **↓** | **↓** | **↓** |
| 71 | Tubulin, beta 5 | **↓** | **↓*** | **↓** | **↓*** |
| 75 | Valosin-containing protein | **↑** | **↑** | **↑*** | **↑*** |
| 76 | Vimentin | **↓** | **↓*** | **↓*** | **↓** |
|  | **Total (including significant) changes** | **15↓, 6↑** | **7↓, 14↑** | **8↓,12↑,1UN** | **13↓, 8↑** |
|  | **Total significant changes** | **2↑*** | **2↓*** | **3↓*, 5↑*** | **1↓*, 2↑*** |

Comparison of mean normalized volumes for proteins from Male to Female mice in the same group (KO, KO 6 hr SP-A, KO 18 hr SP-A, and WT). Higher in female (**↑**), lower in female (**↓**), unchanged (UN), determined to be significant (p<0.05) by t-test (*****).

**Additional File 7**

**Table D. Changes in regulatory/differentiative processes proteins from Male to Female mice in the same group.**

| **Gel No.** | **Protein Name** | **KO** | **KO 6 hr SP-A** | **KO 18 hr SP-A** | **WT** |
| --- | --- | --- | --- | --- | --- |
| 25 | Eukaryotic translation initiation factor 5A | **↓** | **↓** | **↓** | **↑** |
| 41 | Heterogeneous nuclear ribonucleoprotein K | **↓** | **↓** | **↑** | **↑** |
| 42 | High mobility group 1 protein | **↑** | **↓** | **↓** | **↓** |
| 43 | Hnrpf protein | **↓** | **↑** | **UN** | **↓** |
| 44 | Kappa-B motif-binding phosphoprotein | **↑** | **UN** | **↑** | **↑** |
| 52 | Nucleophosmin 1 | **↓** | **↑** | **↑** | **↑** |
| 53 | p50b, Leukocyte specific protein 1 (LSP1) | **↓** | **↓** | **↓** | **↓** |
| 60 | Protein synthesis initiation factor 4A | **↓** | **↑** | **↑*** | **↑*** |
|  | **Total (including significant) changes** | **6↓, 2↑** | **4↓, 3↑, 1UN** | **3↓, 4↑, 1UN** | **3↓, 5↑** |
|  | **Total significant changes** |  |  | **1↑*** | **1↑*** |

Comparison of mean normalized volumes for proteins from Male to Female mice in the same group (KO, KO 6 hr SP-A, KO 18 hr SP-A, and WT). Higher in female (**↑**), lower in female (**↓**), unchanged (UN), determined to be significant (p<0.05) by t-test (*****).

**Additional File 7**

**Table E. Changes in regulation of inflammation proteins from Male to Female mice in the same group.**

| **Gel No.** | **Protein Name** | **KO** | **KO 6 hr SP-A** | **KO 18 hr SP-A** | **WT** |
| --- | --- | --- | --- | --- | --- |
| 5 | Alpha-fetoprotein | **↓** | **↓** | **↓** | **↓** |
| 6 | Annexin A2 | **↓*** | **↓** | **↓** | **UN** |
| 7 | Annexin A4 | **↓** | **↑** | **↓*** | **↓** |
| 16 | Chia protein | **↑** | **↑** | **↓** | **↑** |
| 17 | Chitinase 3-like 3 precursor (Ym1) | **UN** | **↑** | **↑*** | **↑*** |
| 18 | Chitinase-related protein MCRP | **UN** | **↑** | **↑** | **↑** |
| 22 | Coactosin-like 1 | **↓** | **↓** | **↓*** | **↓** |
| 24 | Eno1 protein (Alpha-enolase) | **↓** | **↑** | **↑** | **↑** |
| 25 | Eukaryotic translation initiation factor 5A | **↓** | **↓** | **↓** | **↑** |
| 34 | Heat shock protein 1, beta (HSP90AB1) | **↑*** | **↑** | **↑*** | **↑** |
| 35 | Heat shock protein 5 precursor (GRP78) | **↑*** | **↑** | **↑*** | **↑** |
| 36 | Heat shock protein 65 (HSP60) | **↑** | **↑** | **↑*** | **↑*** |
| 37 | Heat shock protein 8 (HSC70; HSC71) | **↑** | **↑** | **↓** | **↓** |
| 38 | Heat shock protein 90, beta (Grp94), member 1 | **↓** | **↑** | **↑*** | **↑** |
| 39 | Hematopoietic cell specific Lyn substrate 1 | **↓** | **↓** | **↓** | **↓** |
| 40 | Heme-binding protein | **UN** | **↓*** | **UN** | **↓** |
| 42 | High mobility group 1 protein | **↑** | **↓** | **↓** | **↓** |
| 72 | Tyrosine 3/tryptophan 5 -monooxygenase activation protein, ε | **↑** | **↓** | **↑** | **↑** |
| 73 | Tyrosine 3-monooxygenase/tryptophan 5-monooxygenase activation protein, β | **↓** | **↑** | **↑** | **↑** |
| 76 | Vimentin | **↓** | **↓*** | **↓*** | **↓** |
|  | **Total (including significant) changes** | **10↓,7↑,3UN** | **9↓, 11↑** | **10↓, 9↑, 1UN** | **8↓, 11↑, 1UN** |
|  | **Total significant changes** | **1↓*, 2↑*** | **2↓*** | **3↓*, 5↑*** | **2↑*** |

Comparison of mean normalized volumes for proteins from Male to Female mice in the same group (KO, KO 6 hr SP-A, KO 18 hr SP-A, and WT). Higher in female (**↑**), lower in female (**↓**), unchanged (UN), determined to be significant (p<0.05) by t-test (*****).

**Additional File 7**

**Table F. Changes in regulation of all proteins from Male to Female mice in the same group.**

| **Gel No.** | **Protein Name** | **KO** | **KO 6 hr SP-A** | **KO 18 hr SP-A** | **WT** |
| --- | --- | --- | --- | --- | --- |
| 1 | 65-kDa macrophage protein | **↑** | **↑** | **↑** | **↑** |
| 2 | Actin related protein 2/3 complex, subunit 5 | **UN** | **↓** | **↑** | **UN** |
| 3 | Actin-related protein 3 | **↓** | **↑** | **↑** | **↑** |
| 4 | Actr2 protein | **↓** | **↑** | **↓** | **↑** |
| 5 | Alpha-fetoprotein | **↓** | **↓** | **↓** | **↓** |
| 6 | Annexin A2 | **↓*** | **↓** | **↓** | **UN** |
| 7 | Annexin A4 | **↓** | **↑** | **↓*** | **↓** |
| 8 | Anxa5 protein | **↓** | **↓** | **↓*** | **↓** |
| 9 | ArsA arsenite transporter, ATP-binding, homolog 1 | **↓** | **↓** | **↓** | **↑** |
| 10 | Atp5b protein | **↓** | **↓** | **↑** | **↑** |
| 11 | Calpain, small subunit 1 | **↓** | **↑** | **↓** | **↓** |
| 12 | Capping protein (actin filament) muscle Z-line, alpha 2 (CapZ alpha-2) | **↓** | **↓** | **↓*** | **↓** |
| 13 | Capping protein (actin filament) muscle Z-line, beta isoform a (CapZ beta) | **↓** | **↓** | **↓** | **↓** |
| 14 | Cathepsin D precursor | **↓** | **↑** | **↓** | **↓** |
| 15 | Chaperonin subunit 2 (beta) (CCT2) | **↑** | **↑** | **↑** | **↑*** |
| 16 | Chia protein | **↑** | **↑** | **↓** | **↑** |
| 17 | Chitinase 3-like 3 precursor (Ym1) | **UN** | **↑** | **↑*** | **↑*** |
| 18 | Chitinase-related protein MCRP | **UN** | **↑** | **↑** | **↑** |
| 19 | Chloride intracellular channel 1 | **↓** | **↑** | **↑** | **UN** |
| 20 | Chloride intracellular channel 4 (mitochondrial) | **↓** | **↑** | **↓*** | **↓** |
| 21 | CNDP dipeptidase 2 | **↓** | **↑** | **↑** | **↑** |
| 22 | Coactosin-like 1 | **↓** | **↓** | **↓*** | **↓** |
| 23 | EF hand domain containing 2 | **↓** | **↑** | **↓** | **↓** |
| 24 | Eno1 protein (Alpha-enolase) | **↓** | **↑** | **↑** | **↑** |
| 25 | Eukaryotic translation initiation factor 5A | **↓** | **↓** | **↓** | **↑** |
| 26 | Ezrin | **↓** | **↓** | **↓** | **↑** |
| 27 | F-actin capping protein alpha-1 subunit (CapZ alpha-1) | **↓** | **↓** | **↓** | **↓** |
| 28 | Ferritin heavy chain 1 | **↑** | **↑** | **↓** | **↓** |
| 29 | Ferritin light chain 1 | **↓** | **↑** | **↓** | **↓** |
| 30 | Gamma-actin | **↓** | **↓** | **↑** | **↓** |
| 31 | Gelsolin precursor | **↑** | **↑** | **↑** | **↑** |
| 32 | Glucose-6-phosphate dehydrogenase X-linked | **↓** | **↑** | **↑** | **↑** |
| 33 | Guanine deaminase | **↑*** | **↑*** | **↑*** | **↑*** |
| 34 | Heat shock protein 1, beta (HSP90AB1) | **↑*** | **↑** | **↑*** | **↑** |
| 35 | Heat shock protein 5 precursor (GRP78) | **↑*** | **↑** | **↑*** | **↑** |
| 36 | Heat shock protein 65 (HSP60) | **↑** | **↑** | **↑*** | **↑*** |
| 37 | Heat shock protein 8 (HSC70; HSC71) | **↑** | **↑** | **↓** | **↓** |
| 38 | Heat shock protein 90, beta (Grp94), member 1 | **↓** | **↑** | **↑*** | **↑** |
| 39 | Hematopoietic cell specific Lyn substrate 1 | **↓** | **↓** | **↓** | **↓** |
| 40 | Heme-binding protein | **UN** | **↓*** | **UN** | **↓** |
| 41 | Heterogeneous nuclear ribonucleoprotein K | **↓** | **↓** | **↑** | **↑** |
| 42 | High mobility group 1 protein | **↑** | **↓** | **↓** | **↓** |
| 43 | Hnrpf protein | **↓** | **↑** | **UN** | **↓** |
| 44 | Kappa-B motif-binding phosphoprotein | **↑** | **UN** | **↑** | **↑** |
| 45 | Keratin complex 2, basic, gene 8 | **↓** | **↓** | **↑** | **↓** |
| 46 | Keratin type II | **↓** | **↑** | **↑** | **↓** |
| 47 | Krt13 protein | **↓** | **↑** | **UN** | **↓** |
| 48 | Laminin receptor | **↓** | **↑** | **↑** | **↓** |
| 49 | Major vault protein (MVP) | **↑** | **↑** | **↑*** | **↑*** |
| 50 | Microtubule-associated protein, RP/EB family, member 1 | **↓** | **↓** | **↑*** | **↑*** |
| 51 | Myosin light chain, regulatory B-like | **↓** | **↓** | **↑** | **↓** |
| 52 | Nucleophosmin 1 | **↓** | **↑** | **↑** | **↑** |
| 53 | p50b | **↓** | **↓** | **↓** | **↓** |
| 54 | Peroxiredoxin 2 | **↓** | **↓** | **↑** | **↓** |
| 55 | Prolyl 4-hydroxylase, beta polypeptide precursor | **↓*** | **↓** | **↓** | **↓** |
| 56 | Proteasome (prosome, macropain) 28 subunit, alpha | **↓** | **↑** | **↓** | **↓** |
| 57 | Proteasome alpha 1 subunit | **↓** | **↑** | **↑** | **↓** |
| 58 | Protein disulfide isomerase associated 6 (PDI-P5) | **↓** | **↑** | **↑** | **↑** |
| 59 | Protein disulfide-isomerase A3 precursor | **↑** | **↑*** | **↑** | **↑** |
| 60 | Protein synthesis initiation factor 4A | **↓** | **↑** | **↑*** | **↑*** |
| 61 | Purine nucleoside phosphorylase | **↓** | **↑** | **↑** | **↑** |
| 62 | Put. beta-actin (aa 27-375) | **↓** | **↓** | **↓*** | **↓** |
| 63 | Rab GDP dissociation inhibitor beta | **↓** | **↑** | **↑** | **↑*** |
| 64 | Rho GDP dissociation inhibitor (GDI) alpha | **↓** | **↑** | **↑*** | **↑*** |
| 65 | Rho, GDP dissociation inhibitor (GDI) beta | **↑** | **↑** | **↑** | **↑** |
| 66 | Serine (or cysteine) proteinase inhibitor, clade B, member 1a | **↓** | **↓** | **↓** | **UN** |
| 67 | Stathmin | **UN** | **↑** | **↑** | **↓** |
| 68 | Superoxide dismutase 1, soluble | **↓** | **↓** | **↓** | **↓** |
| 69 | Tropomodulin 3 | **↓** | **↓*** | **↓*** | **↓** |
| 70 | Tropomyosin 3, gamma | **↓** | **↓** | **↓*** | **↓** |
| 71 | Tubulin, beta 5 | **↓** | **↓*** | **↓** | **↓*** |
| 72 | Tyrosine 3/tryptophan 5 -monooxygenase activation protein, ε | **↑** | **↓** | **↑** | **↑** |
| 73 | Tyrosine 3-monooxygenase/tryptophan 5-monooxygenase activation protein, β | **↓** | **↑** | **↑** | **↑** |
| 74 | Vacuolar adenosine triphosphatase subunit B | **↓** | **↑** | **↑** | **↑** |
| 75 | Valosin-containing protein | **↑** | **↑** | **↑*** | **↑*** |
| 76 | Vimentin | **↓** | **↓*** | **↓*** | **↓** |
|  | **Total (including significant) changes** | **54↓,17↑,5UN** | **31↓,44↑,1UN** | **32,41↑,3UN** | **36↓,36↑,4UN** |
|  | **Total significant changes** | **2↓*, 3↑*** | **4↓*, 2↑*** | **9↓*, 11↑*** | **1↓*, 10↑*** |

Comparison of mean normalized volumes for proteins from Male to Female mice in the same group (KO, KO 6 hr SP-A, KO 18 hr SP-A, and WT). Higher in female (**↑**), lower in female (**↓**), unchanged (UN), determined to be significant (p<0.05) by t-test (*****).
